# Yao medicine *Amydrium hainanense* suppresses hepatic fibrosis by repressing hepatic stellate cell activation *via* STAT3 signaling

**DOI:** 10.3389/fphar.2022.1043022

**Published:** 2022-12-14

**Authors:** Bingmin Wu, Lan Huang, Yange Wang, Lishan Zeng, Ying Lin, Jingyan Li, Shaogui Wang, Guifang Zhang, Lin An

**Affiliations:** Guangdong Key Laboratory for Translational Cancer Research of Chinese Medicine, Joint Laboratory for Translational Cancer Research of Chinese Medicine of the Ministry of Education of the People’s Republic of China, International Institute for Translational Chinese Medicine, School of Pharmaceutical Science, Guangzhou University of Chinese Medicine, Guangzhou, Guangdong, China

**Keywords:** hepatic fibrosis, Amydrium hainanense, stat3, α-SMA, carbon tetrachloride

## Abstract

**Ethnopharmacological relevance:** Hepatic fibrosis (HF) occurs in response to chronic liver injury and may easily develop into irreversible liver cirrhosis or even liver cancer. *Amydrium hainanense* water extract (AHWE) is a water-soluble component extracted from the Yao medicine *Amydrium hainanense* (H.Li, Y.Shiao & S.L.Tseng) H.Li, which is commonly used for treating inflammatory diseases in folk. Previous evidence suggested that AHWE significantly inhibited hepatic stellate cell activation. However, little is known regarding the therapeutic effect of AHWE in HF and its underlying action mechanism.

**Objective:** Investigation of the therapeutic effect of AHWE in HF and its underlying mechanism.

**Methods:** The therapeutic effect of AHWE was tested *in vivo* using an HF mouse model *via* an intraperitoneal injection of carbon tetrachloride (CCl_4_). Histological evaluation of liver injury and fibrosis were tested by H&E staining and Masson’s trichrome staining. Serum levels of ALT, AST, collagen type I (Col I), and hydroxyproline (HYP) were measured. The mRNA expression of liver fibrotic and inflammatory genes were tested, and the protein levels of alpha smooth muscle actin (α-SMA) and signal transducers and activators of transcription 3 (STAT3) were analyzed. The *in vitro* experiments were conducted using HSC-T6 and RAW264.7 cell lines.

**Results:** Treatment with AHWE significantly reversed histopathological liver damage and liver function abnormalities in CCl_4_ mouse model. Also, the serum levels of ALT, AST, Col I, and HYP in CCl_4_-induced HF mice were improved in AHWE treatment. Further, AHWE showed a remarkable inhibitory effect on the expression of fibrosis markers (*Acta2*, *Col1a1*, and *Col3a1*) and inflammatory factors (*Stat3, Tnfa*, *Il6*, and *Il1b*) induced by CCl_4_. The results of *in vitro* experiments were consistent with those obtained *in vivo*. In addition, it is shown that STAT3 signaling was involved in the anti-fibrotic effects of AHWE as evidenced by STAT3 overexpression.

**Conclusion:** The present study proposed a novel ethnomedicine for HF and suggested the underlying role of STAT3 signaling pathway regulation in this anti-fibrotic effect of the proposed medicine. These findings would serve as solid scientific evidence in support of the development of AHWE as a novel alternative or complementary therapy for HF prevention and treatment.

## 1 Introduction

Hepatic fibrosis (HF) is a reversible and complex wound-healing event secondary to various inflammations and chronic liver injuries (Trautwein et al., 2015). The main characteristic of HF is the excessive extracellular matrix (ECM). When tissue repair occurs in the body, fibrous scars progressively replace the liver parenchyma and distort the vascular architecture of the liver, eventually leading to organ dysfunction (Trautwein et al., 2015). The pathogenic mechanisms of HF may be triggered by several risk factors, such as virus infection, non-alcoholic steatohepatitis (NASH), toxin-related stimuli (e.g., alcohol and chemical), and autoimmune disorders (Parola and Pinzani, 2019). Such damage to liver parenchyma stimulates hepatocytes, Kupffer cells, T cells, and other cells, thereby inducing an inflammatory response, which causes the hepatic stellate cells (HSCs) to transform from a quiescent vitamin A-storing cell lineage to activated myofibroblast-like cells that exhibit contractile properties (Tsuchida and Friedman, 2017). This activation and transformation of HSCs result in the continuous accumulation of alpha-smooth muscle actin (α-SMA), type I and type III collagen (Col Ⅰ, Col Ⅲ), and huge amounts of ECM, causing the wound healing response to become pathogenic (Tsuchida and Friedman, 2017). The end-stage of progressive HF is cirrhosis, which has mortal complications. Liver transplantation is the only effective curative therapy available so far (Trautwein et al., 2015). Fortunately, HF is a reversible process, which allows for preventing the progression of fibrosis and reversing advanced HF to inhibit the further progression of hepatopathy, which ultimately reduces mortality. Therefore, it is imperative to explore and discover effective and safe anti-fibrotic drugs.

The currently known mechanisms of HF are based on HSCs, which are resident mesenchymal cells that could serve as the key cells when exploring and designing novel drug targets for HF. In the injured/inflamed hepatic tissue microenvironment, paracrine signals, such as transforming growth factor β (TGF-β), platelet-derived growth factor (PDGF), connective tissue growth factor (CTGF), and other growth factors, may directly or indirectly induce the transdifferentiate of HSCs into matrix protein-secreting myofibroblasts, which subsequently contribute to the excessive deposition of huge amounts of ECM components, particularly Col Ⅰ and Col Ⅲ (Barrientos et al., 2008). The ECM components accumulate in the space of Disse and form fibrous scars, which leads to sinusoidal capillarization characterized by the loss of endothelial fenestrations, ultimately distorting the normal hepatic architecture (Villesen et al., 2020).

The signal transducers and activators of transcription 3 (STAT3) signaling pathway is associated closely with inflammation and regulates the cytokine signaling cascade (Hillmer et al., 2016). STAT3 plays decisive and context-dependent roles in inflammation, tissue survival, and carcinogenesis (Hillmer et al., 2016). A dysregulated or constitutive activation of STAT3 may lead to adverse functional effects, such as impaired immunity and the development of inflammatory diseases and cancers (Yu et al., 2009). Phosphorylated STAT3 forms homodimers, which translocate to the nucleus, thereby allowing an exchange of signals between the cytoplasm and the nucleus. Upon translocation into the nucleus, p-STAT3 forms a complex with certain coactivators, including p68, and binds to the promoter region of target genes for transcription activation. This signaling pathway is reported to be directly involved in the activation and transdifferentiation of HSCs and the subsequent formation of HF (Xiang et al., 2018). The blockade of the STAT3 signaling pathway has been demonstrated to impede the morphological transdifferentiation of HSCs and reduce the mRNA expression of profibrotic genes (Wang et al., 2018).

*Amydrium hainanense* (H.Li, Y.Shiao & S.L.Tseng) H.Li (AH) is an botanical drug, which is popularly known as China’s unique climbing Fujimoto. The AH plant is recognized as folk medicine and functional food in the Yao region. AH has been traditionally used by the Yao people to prevent or cure various human diseases, particularly hepatitis diseases. Moreover, according to ancient records, AH is effective in the treatment of other types of inflammatory diseases, such as hepatitis, gastritis, gastric ulcer, osteomyelitis, cholecystitis, vasculitis, cellulitis, scabies, and rheumatic arthralgia. Therefore, it was speculated that AH would be effective in the treatment of hepatitis owing to its anti-inflammatory activity. A pre-experiment conducted by our research group revealed that the water extract of AH (AHWE) exhibited anti-fibrogenic effects in mice with liver injury. However, the role of AHWE in liver fibrosis and its underlying mechanism remained to be elucidated. Therefore, the present study aimed to evaluate the potential influence of AHWE on metabolic parameters, liver function, and the pathology of changes in the liver in the experimentally-induced HF model, and subsequently elucidate the possible molecular mechanisms underlying this effect. The study was particularly focused on demonstrating the ability of AHWE to suppress the activation of HSCs and increase the anti-inflammatory response against HF.

## 2 Materials and methods

### 2.1 Chemicals and substances

The botanical drugs used in the experiment were collected from Yangshan County, Qingyuan City, Guangdong Province, and were identified as the whole plant of *Amydrium hainanense* (H.Li, Y.Shiao & S.L.Tseng) H.Li by Zhang Guifang, Associate Professor, Guangzhou University of Traditional Chinese Medicine.

Carbon tetrachloride (C11605036) was purchased from Macklin (Shanghai, China). The enhanced BCA protein assay kit (P0010) and the RIPA lysis buffer (P0013) were purchased from the Beyotime Institute of Biotechnology (Shanghai, China). The primary antibodies, namely, the anti-rabbit α-SMA (14395–1-AP), anti-GAPDH (6004–1-lg), and mouse anti-STAT3 (10253–2-AP), the HEK293-derived recombinant human TGF-β1 (100–21), and the HRP-labeled AffiniPure goat anti-mouse IgG antibody (H + L) (SA00001–1), were purchased from ProteinTech Group Inc. (Rosemont, IL, United States). The primary antibody rabbit anti-phospho-STAT3 (Tyr705) was purchased from Cell Signaling Technology (Danvers, MA, United States). The HRP-labeled goat anti-rabbit IgG antibody (H + L) (BS13278) was purchased from Bioworld Technology (St. Paul, MN, United States). The Alexa fluor 568-conjugated goat anti-rabbit IgG (H + L) antibody was purchased from Abcam (Cambridge, MA, United States). The Alanine transaminase (Kaiser et al., 2005) assay kit (C009–2–1), the aspartate aminotransferase (AST) assay kit (C010–2–1), and the hydroxyproline examination kit (A030–2–1) were purchased from Nanjing Jiancheng Bioengineering Institute (Nanjing, China). The enzyme-linked immunosorbent assay (ELISA) kit Col-I (E-EL-M 0325c) was purchased from Elabscience (Wuhan, China). The primers used in the real-time PCR analysis were obtained from Sangon Biotech (Shanghai, China). The reagents for western blotting were purchased from Bio-Rad (California Hercules, United States). All chemicals and materials used in the experiments were of analytical grade and obtained from local suppliers.

### 2.2 Extraction of Amydrium hainanense water extract (AHWE)

The dried whole plant of *Amydrium hainanense* was pre-soaked in a 20-fold volume of distilled water and then subjected to extraction for 1 h each time using the water decocting method. Re-extraction was performed two times using the same method, followed by combining all obtained extracts and subjecting the final extract to vacuum filtration. After filtration, the excess solvent was evaporated under reduced pressure using a rotavapor to obtain the crude extract. The crude extract was lyophilized to obtain a fine powder, which was considered the final AHWE and was stored at 4 °C until used in experiments.

### 2.3 Analysis of the chemical composition of AHWE using the Q-orbitrap technique

Q-Orbitrap is one of the most recently developed mass spectrometry technologies, which is characterized by high resolution and accurate qualitative determination based on multi-level mass spectrometry data. This analytical method is efficient in the rapid identification of complex components in Chinese herbal medicines (Xie and Chen, 2021) (Dong et al., 2020). In the present study, the Q-Orbitrap high-resolution mass spectrometer was used in the Full MS scan/dd-MS2 (Top N) scan mode to accurately determine the mass number and fragment ion acquisition of the samples. The detection instruments used included Thermo Scientific™, Ultimate™3000RS, Thermo Scientific™, Q Exactive™, ESI source, and the RP-C18 column (150 × 2.1 mm, 1.8 µm). The mass spectrometry conditions were as follows: scan range, 150–2000 m/z; aux gas heater temperature, 350°C; capillary temperature, 300°C; spray voltage, 3.8 kV; and sheath gas pressure, 40 Arb. High purity nitrogen gas (purity ≥99:999%) was used as both aux gas and sheath gas. High purity argon gas (purity ≥99:999%) was used as the collision gas. Full-mass and dd-MS2 data in positive and negative modes were obtained at 70,000 and 17,500 FWHM (full width, half maximum), respectively. The chromatography conditions were as follows: column temperature, 35°C; water phase A), 0.1% aqueous solution of formic acid; organic phase B), acetonitrile solution containing 0.1% formic acid. The elution procedure [A:B (v/v) at time (Villesen et al.)] was as follows: (98:2) at 0 min; (98:2) at 1 min; (80:20) at 5 min; (50:50) at 10 min; (20:80) at 15 min; (5:95) at 20 min; (5:95) at 25 min; (98:2) at 26 min; and (98:2) at 30 min. The sample volume injected was 5.00 µL, and the sample flow rate was 0.30 ml/min. All data were acquired and processed using the CD2.1 software (ThermoFisher), followed by retrieval and comparison in the mzCloud, mzVault, and ChemSpider databases.

### 2.4 Animals and experimental procedures

Eight-week-old male C57BL/6 mice (body weight 20 ± 2 g) were procured from the Laboratory Animal Center of Guangzhou University of Chinese Medicine [SCXK (Guangdong) 2019–0202]. All experimental procedures were conducted in accordance with the Guiding Principles for the Care and Use of Animals (China) and were approved by the Laboratory Animal Ethics Committee of Guangzhou University of Chinese Medicine (No. ZYD-2020–135) on 12 December 2020.

The mice were acclimatized to the temperature-controlled environment (22 ± 2°C) and standard 12-h light/12-h dark photoperiod while having free access to food and water. Subsequently, all mice were randomly assigned to the following five groups (*n* = 8): the control group, the CCl_4_ group, the low-dose AHWE group (40 mg/kg), the moderate-dose AHWE group (80 mg/kg), and the high-dose AHWE group (160 mg/kg). Liver fibrosis was induced in mice *via* an intraperitoneal (i.p.) injection of 20% CCl_4_ [at 1:4 dilution level in olive oil (v/v); dosage: 5 ml/kg body weight] administered twice a week for 4 weeks. The control group mice were administered an i.p. injection of the same volume of olive oil. At the beginning of modeling, all treatment groups received an intragastric administration of AHWE (dissolved in PBS) at the doses of 40, 80, and 160 mg/kg once each day for 4 weeks. On the same day, mice in the control and CCl_4_ groups received equal volumes of PBS using a gastric gavage.

After the experimental period, all mice were subjected to a 12-h fasting period, which was followed by euthanization using 1% pentobarbital sodium (i.p. 50 mg/kg) and retrieval of the liver samples. Next, the liver index was calculated using the formula: the liver index = mice liver weight/mice weight × 100%. The liver lobe was fixed in 4% paraformaldehyde for histological evaluation, while the other liver portions were stored at –80°C until used for further examination.

### 2.5 Serum biochemical assays

The blood samples were left undisturbed at room temperature for 1 h, followed by centrifugation at 3,000 rpm for 15 min at 4°C. The obtained sera were subjected to the evaluation of alanine aminotransferase (Kaiser et al., 2005) and aspartate aminotransferase (AST) levels using the protocols provided in the respective kits.

### 2.6 Enzyme-linked immunosorbent assay (ELISA)

The liver tissue samples were homogenized in PBS, on ice, followed by centrifugation at 5,000 *g* for 10 min at 4 °C. The obtained tissue supernatant was subjected to the evaluation of the expression of inflammatory indicators, including Tnfa, Il1β, and Il6, using ELISA kits following the specific instructions provided by the manufacturer.

### 2.7 Liver histopathology and immunohistochemical examination

In order to assess morphological changes in the liver tissue, the liver tissues were treated with 4% paraformaldehyde and embedded in paraffin. Subsequently, 5 µm sections were excised from the paraffin blocks and mounted on slides. The paraffin-embedded tissue samples were stained with the hematoxylin and eosin (H&E) stain for histopathological examination or with Masson’s trichrome stain for collagen level determination. The stained sample slides were examined under a digital scanner (E100, Nikon Corporation). Meanwhile, the paraffin-embedded tissues for all groups were subjected to immunohistochemical staining for STAT3 and visualized using a section digital scanner (E100, Nikon Corporation).

### 2.8 Cell culture and AHWE treatment

The rat hepatic stellate cell line T6 (HSC-T6) was kindly provided by the Zhejiang Cancer Hospital (Zhejiang, China). The cells were cultured in Dulbecco’s modified Eagle’s medium (DMEM, Thermo Fisher Scientific, MA, United States) supplemented with 10% fetal bovine serum (FBS, Thermo Fisher Scientific, MA, United States), 100 U/mL of streptomycin, and 100 U/mL of penicillin at 37°C inside a humidified incubator with 5% CO_2_ and 95% air. Upon reaching the desired confluence, the cells were washed two times with PBS and cultured in a fresh serum-free medium prior to subsequent experiments.

In order to establish the HSCs injury model, the HSC-T6 cells were treated with 10 ng/ml of transforming growth factor beta 1 (TGF-β1) for 1 h for stimulation. Subsequently, AHWE was added directly at different doses (0.25, 0.5, and 1 mg/ml) to the DMEM supernatant containing TGF-β1, followed by 24 h of incubation. The inflammatory cell model was established by treating the RAW264.7 cell line with 1 μg/ml of lipopolysaccharide (LPS) for 1 h. Afterward, the drug solution was added directly at different doses (0.25, 0.5, and 1 mg/ml) to the DMEM supernatant containing LPS, followed by 24 h of incubation.

### 2.9 Cell viability assay

Cellular viability was evaluated using the CCK-8 assay kit (GLPBIO, California, United States). The HSC-T6 cells were cultured in 96-well culture plates and then treated with different concentrations of AHWE ranging from 0 to 2.5 mg/ml for 24 h. Next, the CCK-8 solution (10 µL) was added to each well, and the absorbance was measured after 1 h of incubation using a microplate reader (Thermo Varioskan LUX, MA, United States) at 450 nm. Each experiment was conducted in triplicate for each group. Percent viability was calculated as the absorbance of the cells treated with different doses of AHWE relative to that of the control cells.

### 2.10 Immunofluorescence staining

RAW264.7 cells cultured on confocal dishes were treated with the corresponding stimuli. After washing with PBS, the cells were treated with 4% paraformaldehyde for 15 min at room temperature for fixing and then permeabilized with PBS containing 0.1% Triton X-100 for another 10 min at room temperature. Next, the cells were blocked by exposure to 10% goat serum for 30 min at room temperature, followed by overnight incubation at 4°C with the mouse antibody against STAT3 (for STAT3 subcellular detection) in 10% goat serum. After the incubation, the dishes were washed and incubated with Alexa Fluor 594-conjugated secondary antibody in the dark at room temperature for 1 h. The nuclei were detected through 10 min of staining with the DAPI solution in the dark at room temperature. Finally, the dishes were washed and then visualized and photographed under a Zeiss LSM 510 laser confocal fluorescence microscope (Carl Zeiss, Oberkochen, Germany).

### 2.11 Quantitative real-time polymerase chain reaction (RT-qPCR)

Total RNA was extracted from cells or liver tissues using Trizol Reagent (Accurate Biotechnology, Human, China) according to the manufacturer’s instructions. The purity and concentration of the extracted RNA sample were measured using NanoDrop 2000 spectrophotometer from ThermoFisher Scientific (Waltham, MA, United States). Subsequently, 1 ng of the extracted total RNA was used for synthesizing cDNA using the *Evo M-MLV* RT kit, and the gDNA Clean was used for conducting RT-qPCR (Accurate Biotechnology, Human, China). The RT-qPCR experiment was conducted in triplicate for each cDNA sample using the SYBR Green Premix *Pro Taq* HS qPCR kit (Accurate Biotechnology, Human, China) and the gene-specific primer pairs for Gapdh, Col1a1, Col3a1, Il1β, Il6, Tnfa, and Acta2. A QuantStudio five Real-Time PCR instrument (ThermoFisher Scientific, MA, United States) was employed for PCR amplification under the following reaction conditions: 40 cycles of enzyme activation at 95°C for 30 s, denaturation at 95°C for 5 s, and annealing and extending at 60°C for 30 s. The primer sequences used are presented in Table 1. The mRNA expression was calculated using the 2^−ΔΔCT^ method and GAPDH as the internal control.

**TABLE 1:** Sequences of primers used for RT-qPCR.

Species	Primers	Sequences
*Mus musculus*	*Gapdh*-forward	GCC​TCG​TCC​CGT​AGA​CAA​AA
	*Gapdh*-reverse	TAC​GGC​CAA​ATC​CGT​TCA​CA
	*Acta2*-forward	GAA​GCT​CGT​TAT​AGA​AAG​AGT​GG
	*Acta2*-reverse	TCA​GGG​AGT​AAT​GGT​TGG​AAT
	*Col1a1*-forward	TTC​TCC​TGG​CAA​AGA​CGG​AC
	*Col1a1*-reverse	CGG​CCA​CCA​TCT​TGA​GAC​TT
	*Col3a1*-forward	ACG​TAA​GCA​CTG​GTG​GAC​AG
	*Col3a1*-reverse	CAG​GAG​GGC​CAT​AGC​TGA​AC
	*Il1β*-forward	TGC​CAC​CTT​TTG​ACA​GTG​ATG
	*Il1β*-reverse	TGA​TGT​GCT​GCT​GCG​AGA​TT
	*Il6*-forward	GТССТТССТАССССААТТССА
	*Il6*-reverse	CGC​ACT​AGG​TTT​GCC​GAG​TA
	*Tnfa*-forward	ATG​GCC​TCC​CTC​TCA​TCA​GT
	*Tnfa*-reverse	TTT​GCT​ACG​ACG​TGG​GCT​AC
	*Stat3*-forward	TGT​CAG​ATC​ACA​TGG​GCT​AAA​T
	*Stat3*-reverse	GGT​CGA​TGA​TAT​TGT​CTA​GCC​A
*Rattus norvegicus*	*Gapdh*-Forward	AGT​GCC​AGC​CTC​GTC​TCA​TA
	*Gapdh*-Reverse	GAT​GGT​GAT​GGG​TTT​CCC​GT
	*Acta2*-forward	CAT​CCG​ACC​TTG​CTA​ACG​GA
	*Acta2*-reverse	GTC​CAG​AGC​GAC​ATA​GCA​CA
	*Col1a1*-forward	GTGCGATGGCGTGCTATG
	*Col1a1*-reverse	ACT​TCT​GCG​TCT​GGT​GAT​ACA
	*Col3a1*-forward	AGA​TGC​TGG​TGC​TGA​GAA​GAA​AC
	*Col3a1*-reverse	GCT​GGA​AAG​AAG​TCT​GAG​GAA​GG

### 2.12 Plasmid transfection

The STAT3 plasmid was constructed using pEGFP-N3. The plasmid construction was confirmed through DNA sequencing, which was conducted in Sangon Biotech Co. Ltd. (Shanghai, China). The constructed plasmid was then transiently transfected into HSC-T6 and RAW264.7 cells using Lipofectamine 2000 reagent (Invitrogen, Carlsbad, CA, United States) according to the manufacturer’s instructions. After 24 h, the cells were cultured in a fresh medium and used for subsequent experiments.

### 2.13 Western blotting analysis

The liver tissue and cultured cell lysates were prepared, on ice, using RIPA lysis buffer containing a protease inhibitor cocktail. The concentration of proteins in the lysates was determined using the BCA protein assay kit (Pierce, United States). Equal amounts of proteins were separated using 10%∼12% sodium dodecyl sulfate-polyacrylamide gel electrophoresis (SDS-PAGE) run at 55 V for 30 min and then at 120 V for 60 min. The separated proteins were transferred from the gels to methanol-presoaked polyvinylidene fluoride (PVDF) membranes under the action of a constant current of 350 A for 70 min. Next, the membranes were blocked using 5% skimmed milk (FUJIFILM Wako Pure Chemical Corporation, Fuji, Japan), followed by incubation at room temperature for 2 h and subsequent washing with Tris-buffered saline Tween (TBST). The membranes were then incubated overnight at 4°C with primary antibodies against GAPDH, α-SMA, and STAT3 (used at 1:1000 dilution), followed by incubation with the appropriate horseradish peroxidase-conjugated secondary antibody against rabbit or mouse IgG (used at 1:10000 dilution). The band intensities were developed using ECL enhanced chemiluminescence (Yeasen, Shanghai, China) and quantified using Image-ProPlus 6.0 software (Rockville, MD, United States).

### 2.14 Statistical analysis

All experiments were randomized and blinded. The result data were expressed as the mean ± SD (standard deviation) of a minimum of three independent experiments. The result data were analyzed statistically by performing Dunnett’s test and one-way ANOVA using GraphPad Prism version 7.0 (San Diego, CA, United States). The statistical significance threshold was set at *p* < 0.05.

## 3 Results

### 3.1 Chemical composition of AHWE

**FIGURE 1:**
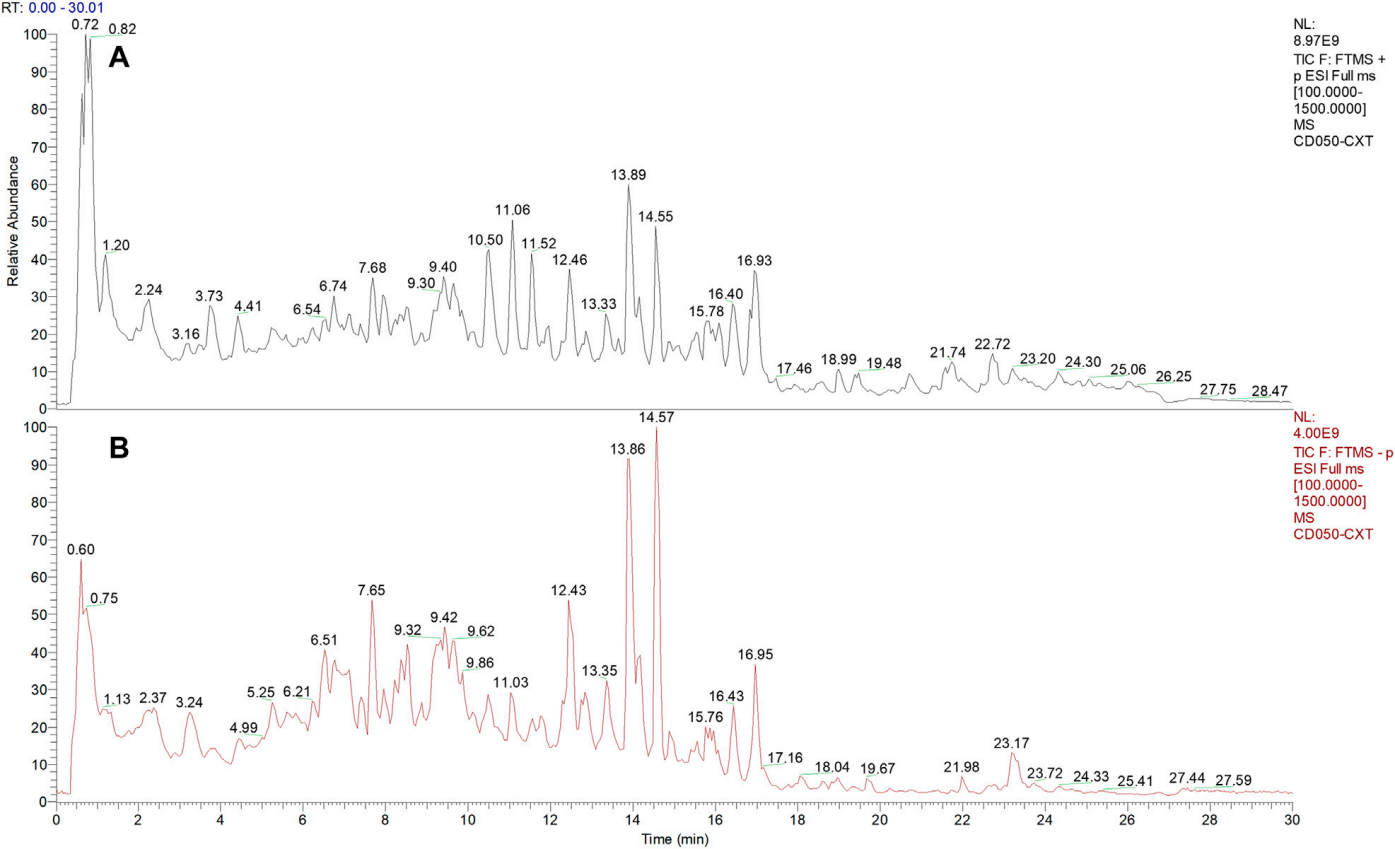
Chemical composition of AHWE. **(A)** Positive mode. **(B)** Negative mode.

The phytochemical compounds present in AHWE were isolated and characterized using the Q-Orbitrap technique. CD2.1 (Thermo Fisher) was employed to preliminarily organize the collected high-resolution liquid quality data, which was followed by searching the databases mzCloud, mzVault, ChemSpider for data comparison. The database search revealed 10 compounds with the mzCloud score of 80 or higher. The total ion chromatogram of AHWE is depicted in Figure 1. The characterization results for the compounds (salsolinol, α-eleostearic acid, corchori fatty acid F, l-phenylalanine, trans-3-Indoleacrylic acid, 9-oxo-10E), 12 (E)-octadecadienoic acid, (+/–)12 (13)-DiHOME, (–)-caryophyllene oxide, citric acid, and trans-4-indoleacrylic acid) are presented in Table 2.

**TABLE 2:** Identification of the chemical constituents in AHWE.

NO.	m/z	RT Villesen et al. (2020)	Formula	Peak area	Identification
1	162.0677	0.673	C_10_H_13_NO_2_	292352457	Salsolinol
2	278.2234	16.994	C_18_H_30_O_2_	284337100	α-Eleostearic acid
3	328.2246	13.357	C_18_H_32_O_5_	205948422	Corchorifatty acid F
4	148.0523	1.197	C_9_H_11_NO_2_	126521147	l-Phenylalanine
5	187.0631	2.266	C_11_H_9_NO_2_	124378283	trans-3-Indoleacrylic acid
6	294.2186	13.924	C_18_H_30_O_3_	112510287	9-Oxo-10E),12(E)-octadecadienoic acid
7	314.2452	15.999	C_18_H_34_O_4_	108470966	(+/-)12 (13)-DiHOME
8	220.182	12.501	C_15_H_24_O	103084519	(-)-Caryophyllene oxide
9	192.0263	0.742	C_6_H_8_O_7_	82592598	Citric acid
10	278.1512	16.094	C_16_H_22_O_4_	34670922	trans-4-Indoleacrylic acid

### 3.2 AHWE improved the CCl4-induced pathological damage in the liver of HF mice

**FIGURE 2:**
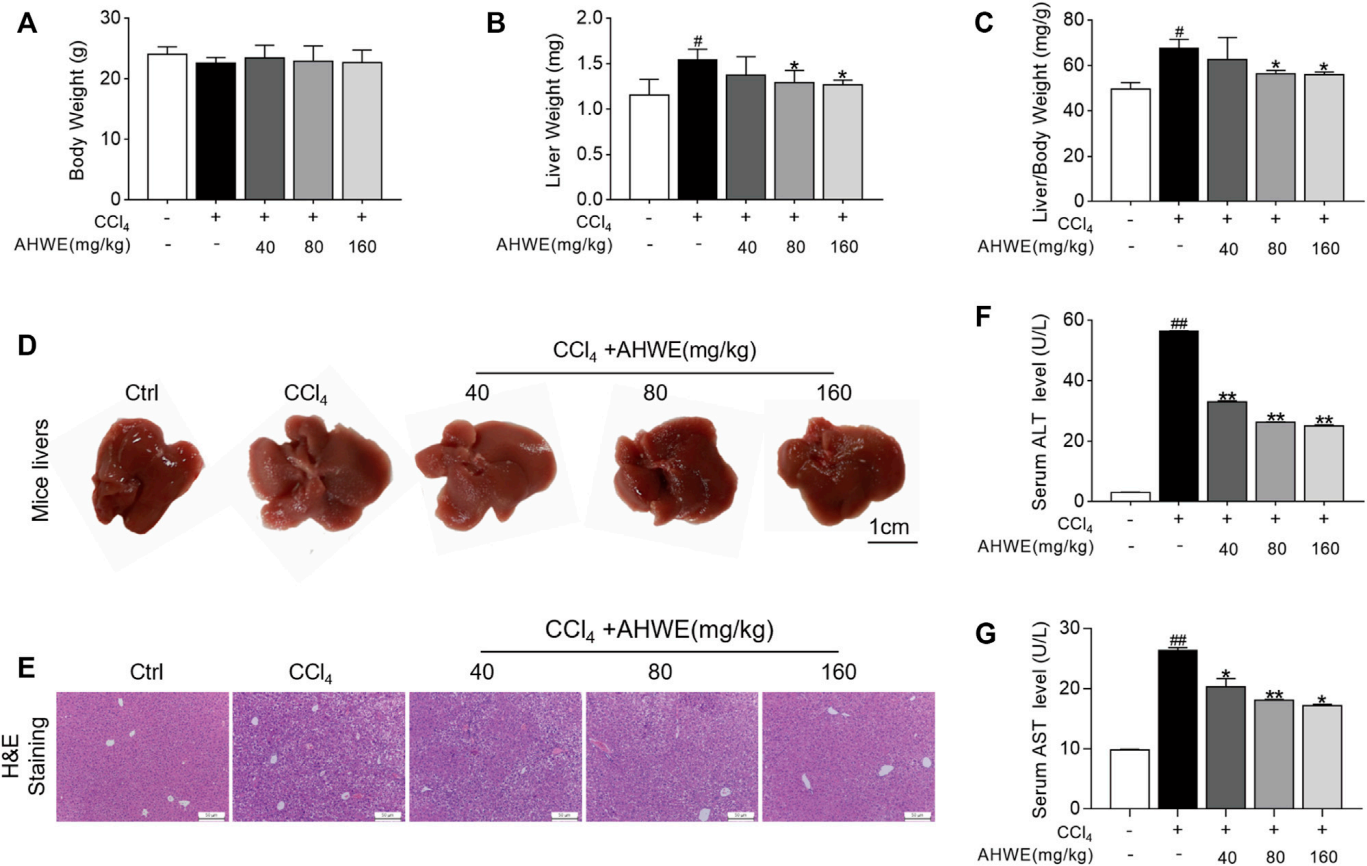
AHWE improved liver pathological damage of CCl_4_-induced HF mice. **(A–C)** Body weight, liver weight and liver/body weight index (%) in mice; **(D)** Gross examination of livers in mice (scale bar: 1 cm); **(E)** Histological examination of liver sections stained with H&E staining (scale bar: 100 μm); **(F,G)** Determination of the serum levels of ALT and AST with assay kits. For the statistics of each panel in this figure, data are expressed as mean ± SD (*n* = 3). #*p* < 0.05 and ##*p* < 0.01 vs. control group; ∗*p* < 0.05 and ∗∗*p* < 0.01 vs. CCl_4_ treatment.

In order to evaluate the effects of AHWE on the CCl_4_-induced HF in mice, the body weight, liver weight, liver index, hepatic histopathological changes, and plasma markers of these mice were monitored (Figure 2). As depicted in Figure 2A, the control group mice exhibited progressive weight gain compared to the CCl_4_ group. In the AHWE-treated mice, no significant difference was observed in the body weight, and the liver weight and liver coefficient values were rescued (Figures 2B, C). Moreover, the morphological changes in the dissected liver samples caused due to CCl_4_ administration were notably improved upon AHWE administration (Figure 2D). These findings were confirmed in the H&E staining results. The H&E stained liver tissue samples from the control group revealed normal hepatocytes with a prominent nucleolus and a complete cytoplasmic pattern and structure of liver lobules. On the contrary, the samples from the CCl_4_ administration group exhibited damaged hepatocytes, inflammatory cell infiltration, necrosis, and steatosis, in addition to visible fat vacuoles. However, these pathological changes in the CCl_4_ group mice were evidently improved in the liver samples from the AHWE-treated mice (Figure 2E). AST and ALT are considered important markers of CCl_4_ hepatotoxicity. In the present study, it was revealed that CCl_4_ administration significantly elevated the levels of ALT and AST (Figures 2F, G). After treatment with AHWE, the liver function of mice demonstrated a trend of recovery. Collectively, the above results suggested that AHWE attenuated the CCl_4_-induced liver pathological damage. Accordingly, based on the hepatoprotective effects of AHWE, it was speculated that AHWE possibly contributes to the prevention of liver fibrosis.

### 3.3 AHWE exhibited an anti-fibrotic effect in CCl_4_-induced HF mice

**FIGURE 3:**
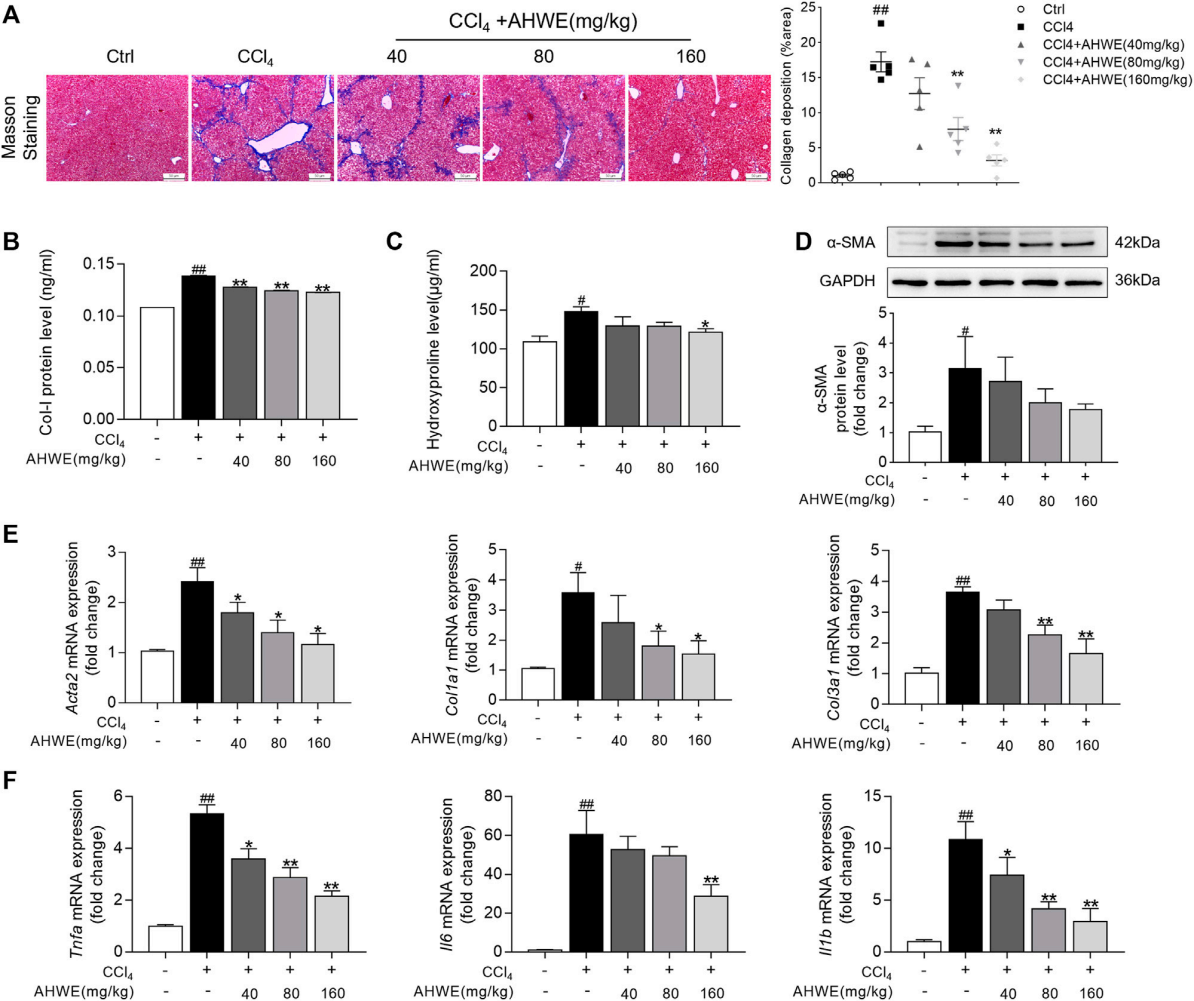
AHWE exhibited antifibrotic effect in CCl_4_-induced HF mice. **(A)** Observation and semi-quantification the collagenous deposition of liver sections stained with the Masson’s trichrome (scale bar: 100 μm); **(B)** Enzyme-linked immunosorbent assays for type I collagen of each group; **(C)** Measurement of hydroxyproline levels by a hydroxyproline assay kit; **(D)** Western blot analyses of α-SMA protein with densitometry. Quantified western blot results of α-SMA proteins; **(E–F)** Q-PCR analyses of *Acta2*, *Col1a1*, *Col3a1*, *Tnfa*, *Il6*, *Il1β* in liver tissues. For the statistics of each panel in this figure, data are expressed as mean ± SD (*n* = 3). #*p* < 0.05 and ##*p* < 0.01 vs. control group; ∗*p* < 0.05 and ∗∗*p* < 0.01 vs. CCl_4_ treatment.

In order to further evaluate the potential of AHWE in reducing liver fibrosis, Masson’s trichrome staining was performed. According to the staining results, AHWE treatment dramatically mitigated collagen deposition, connective tissue hyperplasia, and pseudo-lobule formation induced by CCl_4_ administration (Figure 3A, left panel). The fibrosis indicated in blue in Masson sections was semi-quantified (Figure 3A, right panel). Consistent with these observations, the ELISA results in the present study revealed that AHWE reduced the levels of Col Ⅰ in CCl_4_-induced HF mice (Figure 3B). Similar results were observed in the detection of hydroxyproline (HYP), a major component of all types of fibrillar collagen (Figure 3C). Alpha smooth muscle actin (α-SMA) is considered a marker of the activation of HSCs in the pathological process of HF (Tarbit et al., 2019). The quantified result data from the western blot analysis conducted in the present study revealed that CCl_4_ administration increased the expression of α-SMA in livers, while the treatment with AHWE inhibited the expression of this protein in CCl_4_-induced HF mice (Figure 3D). In addition, fibrotic genes, such as *Acta2*, *Col1a1*, and *Col3a1*, were analyzed using RT-qPCR, which revealed an evident increase in the mRNA levels of all three genes in the CCl_4_ group compared to the control group. However, in the AHWE treatment groups, these levels were markedly downregulated compared to the CCl_4_ group (Figure 3E). Considering the crucial role of inflammation in HF, the levels of common inflammatory factors, such as *Tnfa*, *Il6*, and *Il1β*, were evaluated. It was observed that compared to the control group, the CCl_4_ group exhibited elevated mRNA expression levels of cytokines, while this effect was abolished after AHWE treatment (Figure 3F). These observations confirmed the anti-fibrotic effect of AHWE in CCl_4_-induced HF mice and that AHWE could suppress the inflammatory response during liver fibrosis.

### 3.4 AHWE inhibited hepatic stellate cell activation and suppressed inflammation in cellular experiments

**FIGURE 4:**
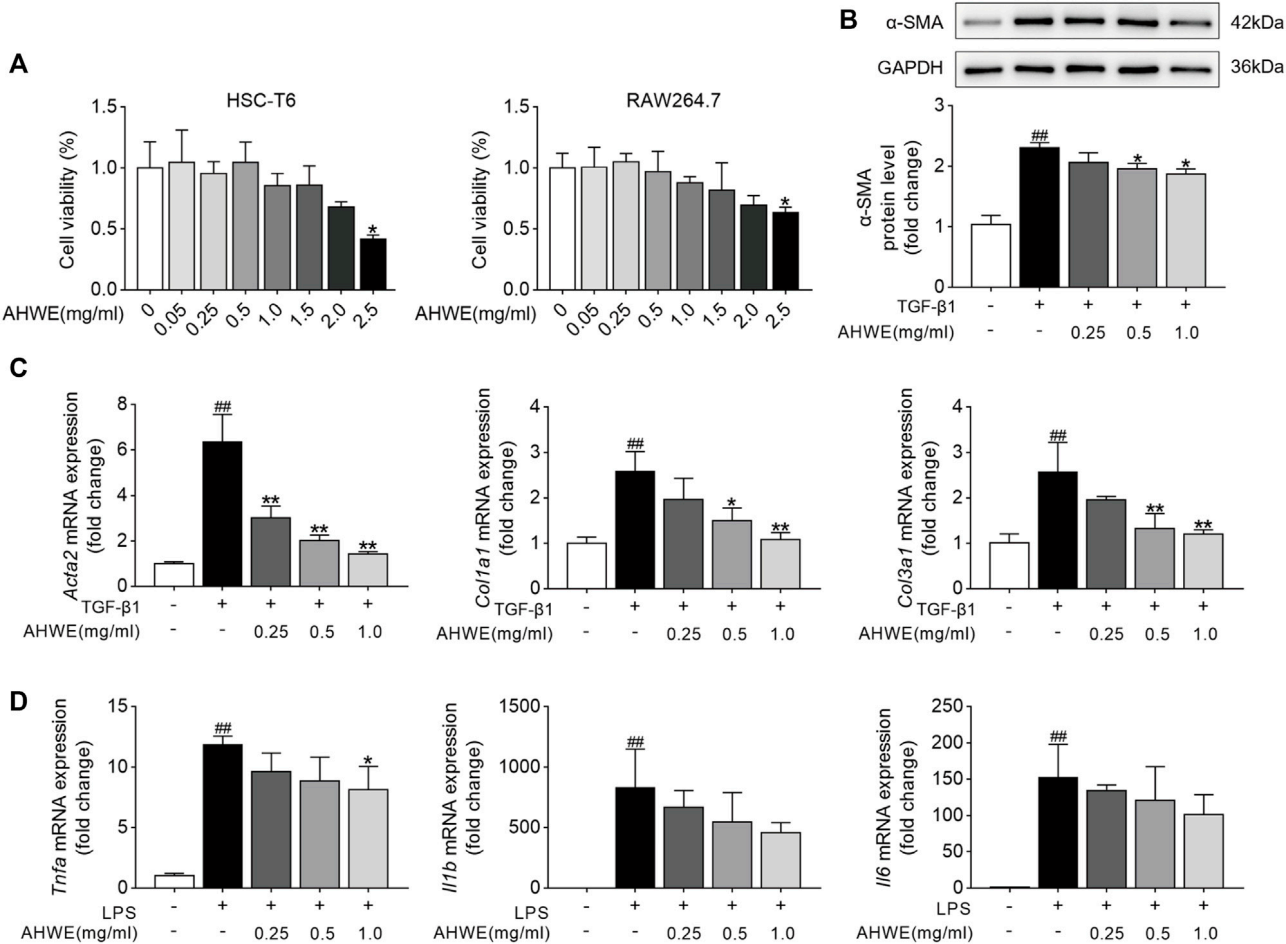
AHWE inhibited hepatic stellate cells activation and suppressed the inflammation in cell experiments. **(A)** The cytotoxicity of AHWE in the HSC-T6 cells and RAW 264.7 cells. Treatment with AHWE at different concentrations (0.005, 0.025,0.05,0.25,1.25, 2.5 mg/ml) for 24 h. Cell viability was detected by performing a CCK-8 assay (*n* = 6). **(B)** The expression of the α-SMA was assessed by Western Blot (*n* = 3). GAPDH was used as a loading control. Representative images of three independent experiments are shown. **(C,D)** The mRNA expression of different fibrosis and inflammatory factors with or without interference with AHWE in TGF-β1-induced HSC-T6 cells (*n* = 4) or RAW264.7 cell line. Data are expressed as the mean ± SD. #*p* < 0.05 and ##*p* < 0.01 vs. control group; ∗*p* < 0.05 and ∗∗*p* < 0.01 vs. TGF-β1 treatment or LPS treatment.

Inspired by the findings of *in vivo* experiments, the anti-fibrotic effects of AHWE and the underlying mechanisms were evaluated *in vitro* as well. In these evaluations, HSC-T6 cells and RAW264.7 cells were used. First, the potential cytotoxicity of AHWE in the above 2 cell types was evaluated, and the statistically analyzed results are presented in Figure 4A. As evident, 2.5 mg/ml AHWE significantly affected cell viability in both cell types (*p* < 0.05), while AHWE in the concentration range of 0.005–1.25 mg/ml was almost non-toxic to the 2 cell types (although with no statistically significant difference). Therefore, in the next experiment, three appropriate doses of AHWE (0.25, 0.5, and 1 mg/ml) were tested to exclude the possibility of AHWE being cytotoxic and thereby causing the inhibition of HSC activation and inflammation.

Furthermore, consistent with the *in vivo* results, AHWE downregulated the α-SMA protein levels induced by TGF-β1 (Figure 4B). In addition, the differentially expressed genes associated with the fibrosis process in HSC-T6 cells, including *Acta2*, *Col1a1*, and *Col3a1*, were identified using the RT-qPCR method. The genes related to the inflammatory process, such as *Tnfa, Il6*, and *Il1β*, were also identified using the same method. The results revealed that TGF-β1 stimulation markedly increased the mRNA expression levels of *Acta2*, *Col1a1*, and *Col3a1* in HSC-T6 cells, while LPS stimulation upregulated the mRNA expression levels of *Tnfa*, *Il6*, and *Il1β* in RAW264.7 cells (Figures 4C, D). As expected, these effects were significantly reversed (*p* < 0.05) upon AHWE treatment in a dose-dependent manner (Figures 4C, D). Collectively, these results suggested that this botanical drug exerted a remarkable inhibitory effect on hepatic stellate cell activation and inflammatory responses.

### 3.5 STAT3 signaling pathway could be involved in the regulation of the anti-fibrotic responses of AHWE

**FIGURE 5:**
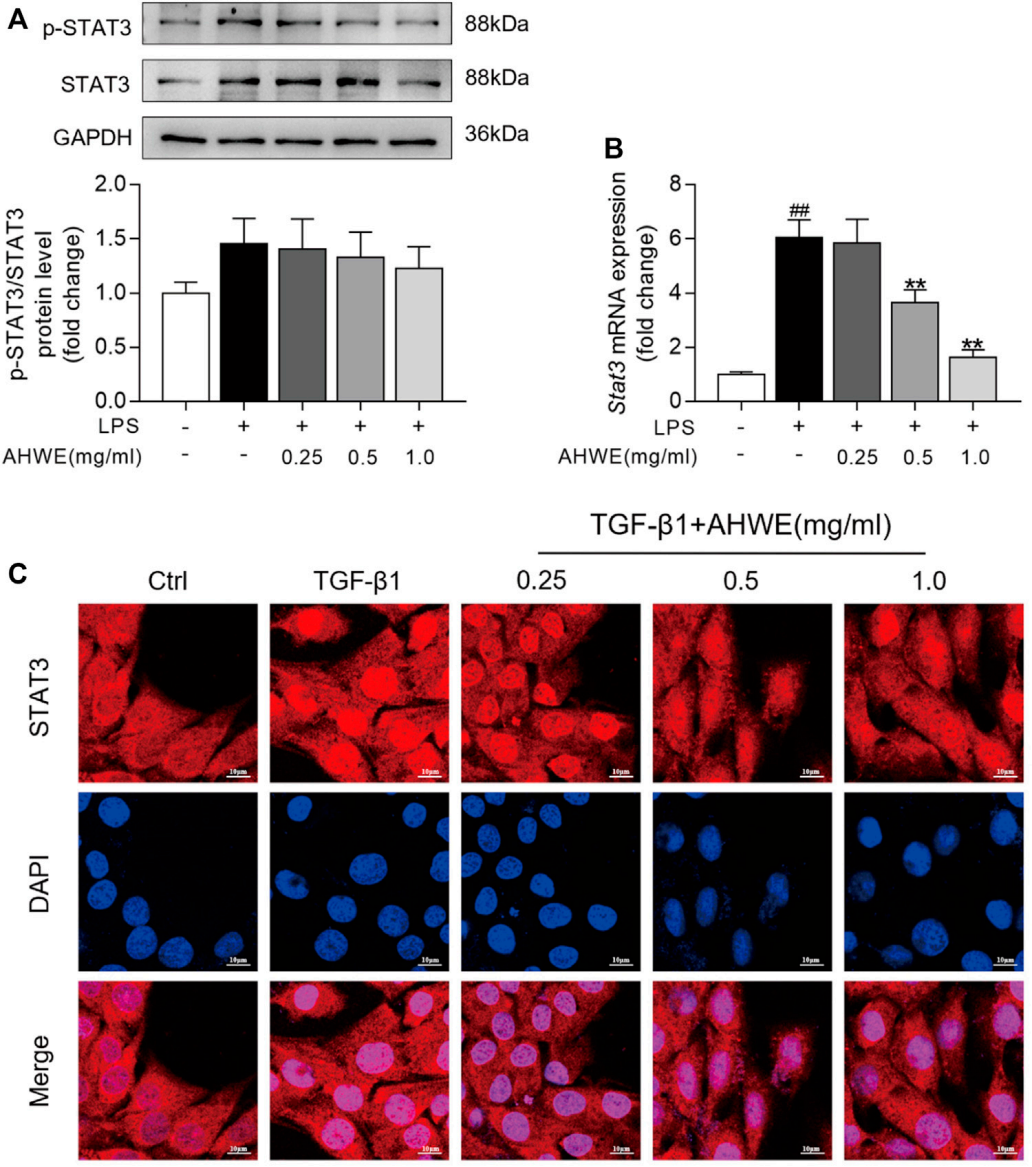
STAT3 signaling pathway might be involved into the regulation of antifibrotic responses by AHWE. **(A)** Western Blotting for phosphorylated STAT3 and total STAT3; Densitometric analyses of bands were quantified, and data expressed as fold of control normalized to GAPDH. **(B)** Expression of *Stat3* mRNA was determined by Q-PCR. The results are representative of at least 3 independent experiments. **(C)** Confocal scanning images of STAT3 nucleus trafficking in HSC-T6 cells stimulated with TGF-β1 (10 ng/ml) for 24 h (*n* = 3), scale bar = 10 μm. Data are expressed as mean ± SD (*n* = 3). #*p* < 0.05 and ##*p* < 0.01 vs. control group; ∗*p* < 0.05 and ∗∗*p* < 0.01 vs. TGF-β1 treatment.

Inflammation serves as a fuel that accelerates liver cell proliferation and tissue regeneration, thereby playing a pivotal role in the pathogenesis of liver fibrosis (Tanwar et al., 2020). Accumulating evidence suggests that STAT3 is closely associated with the occurrence and development of liver fibrosis caused by various factors (Xiang et al., 2018) (Wang et al., 2018). Persistent activation of STAT3 exerts a pro-inflammatory effect, which leads to various pathological manifestations in liver fibrosis. In order to explore whether AHWE exerts an effect on STAT3, the protein levels of STAT3 and p-STAT3 in RAW 264.7 cells were determined using western blotting. The blot images and the quantified data, depicted in Figure 5A, revealed that the trend of increase in the p-STAT3 to STAT3 ratio was higher in the LPS stimulation group than in the control group. AHWE decreased the levels of total and phosphorylated STAT3. In addition, the mRNA expression levels of *Stat3* in RAW264.7 cells were determined using the RT-qPCR method, and it was revealed that the mRNA expression of *Stat3* in the RAW cells stimulated by LPS was significantly increased compared to the control group (Figure 5B). The presence of AHWE relieved this effect in a dose-dependent manner (*p* < 0.05). STAT3 is located mainly in the cytoplasm of resting cells and, in response to activation, translocates to the nucleus to play its role *via* transcriptional regulation. TGF-β1 was observed to increase the STAT3 expression in the nucleus, while STAT3 protein in the cytoplasm was concordantly reduced, which indicated the translocation of STAT3 to the nucleus (Figure 5C). The confocal microscopy observations confirmed the functional role of AHWE in the inhibition of STAT3 activation (Figure 5C). The above experimental evidence indicated that STAT3 signaling pathway could be involved in the regulation of anti-fibrotic responses.

### 3.6 AHWE restrained HSC cell activation through suppression of the STAT3 signaling pathway

**FIGURE 6:**
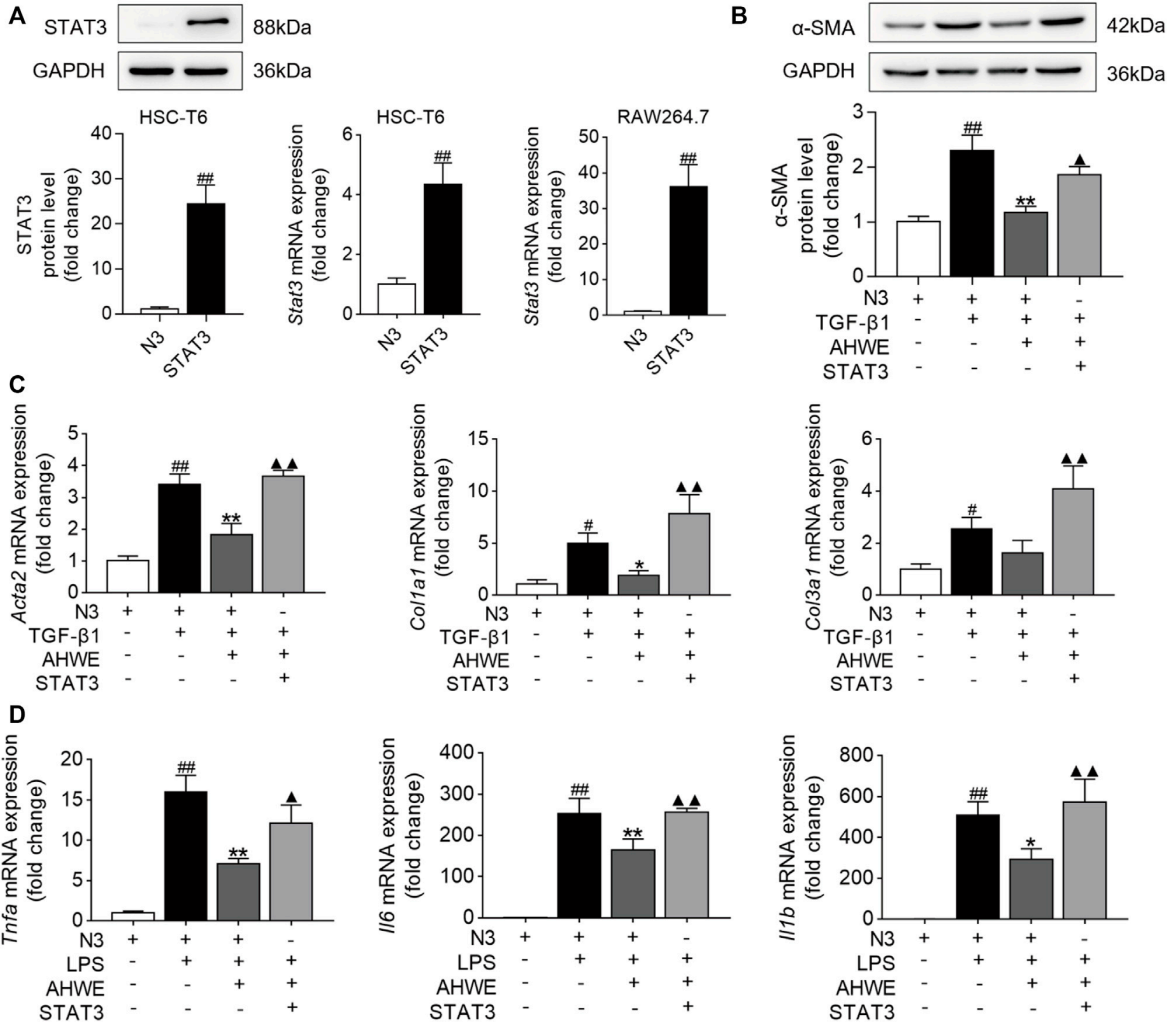
AHWE restrained HSCs activation *via* the suppression of STAT3 signaling pathway. STAT3 were transiently overexpression in HSC-T6 cells and RAW264.7 cells with pEGFP-STAT3 plasmid for 24 h **(A)** STAT3 transfection efficiency verified by western blot and RT-qPCR; **(B)** Western blot showing the protein expressions of α-SMA; **(C,D)** RT-qPCR analysis showing the expressions of genes *Acta2*, *Col1a1*, *Col3a1*, *Tnfa*, *Il6*, and *Il1β*. The results are representative of at least 3 independent experiments. For the statistics of each panel in this figure, data are expressed as mean ± SD (*n* = 3). #*p* < 0.05 and ##*p* < 0.01 vs. control group; ∗*p* < 0.05 and ∗∗*p* < 0.01 vs. model group; ^▲^ *p* < 0.05 and ^▲▲^ *p* < 0.01 vs. AHWE group.

The potential role of the STAT3 signaling pathway in the AHWE regulatory effect on the anti-fibrosis response was further ascertained experimentally using the pEGFP-STAT3 plasmid or the empty N3 vector. The results of western blotting and RT-qPCR verified the successful overexpression of the Stat3 gene in HSC-T6 cells and RAW264.7 cells. As depicted in Figure 6A, STAT3 was overexpressed upon STAT3 plasmid transfection. Next, western blotting was performed to evaluate the levels of α-SMA in the HSC-T6 cells transfected with STAT3 plasmid, and the result data were quantified. As depicted in Figure 6B, the expression levels of this important marker protein were significantly increased upon stimulation with TGF-β1 compared to stimulation with the empty N3 vector. This effect was significantly improved upon treatment with AHWE. The protein expression levels of the fibrosis marker α-SMA were also significantly increased upon STAT3 overexpression, suggesting that STAT3 suppressed the activation of HSCs. In addition, the mRNA expression levels of the fibrosis markers *Acta2*, *Col1a1*, and *Col3a1* and those of the inflammatory markers *Tnfa*, *Il6*, and *Il1β* in the HSC-T6 cells transfected with the STAT3 plasmid were determined (Figures 6C, D). It was revealed that the effect of downregulation of these genes due to AHWE treatment was significantly reversed upon STAT3 overexpression (Figures 6C, D). These findings further elucidated that the *in vitro* anti-fibrotic effect of AHWE was indeed mediated by the STAT3 signaling pathway.

### 3.7 AHWE could regulate the STAT3 signaling pathway to attenuate HF

**FIGURE 7:**
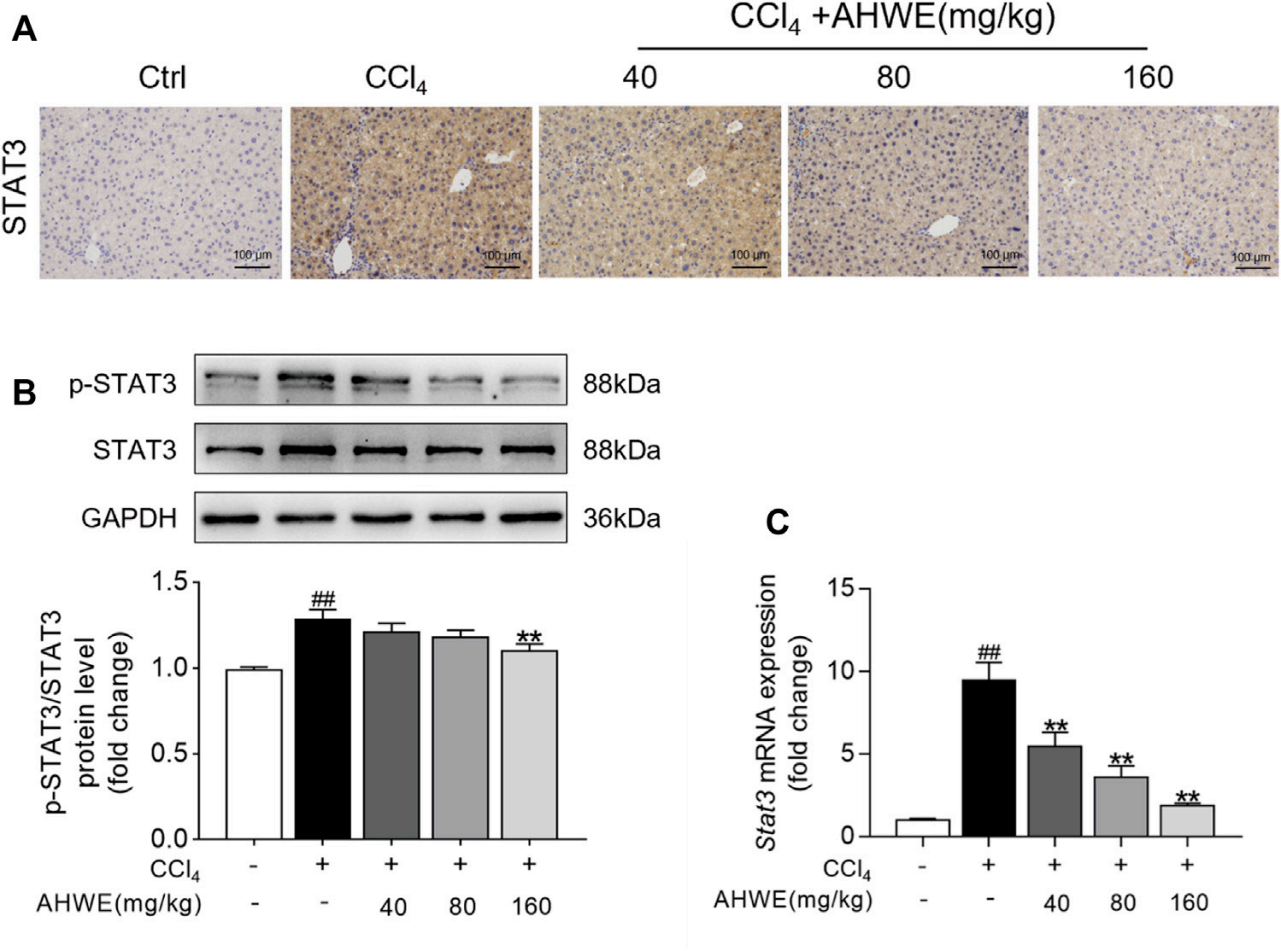
AHWE was able to regulate STAT3 signaling pathway to attenuate HF. **(A)** The representative photomicrographs of immunohistochemistry staining of STAT3 in paraffin-embedded liver samples. **(B)** Western blot and densitometric analysis of STAT3, p-STAT3 and GAPDH; **(C)** Q-PCR for STAT3. For the statistics of each panel in this figure, data are expressed as mean ± SD (*n* = 3). #*p* < 0.05 and ##*p* < 0.01 vs. control group; ∗*p* < 0.05 and ∗∗*p* < 0.01 vs. CCl_4_ group.

The above-stated findings of *in vitro* experiments verified that AHWE could restrain the activation of HSCs by inhibiting the STAT3 signaling pathway. However, the underlying consistent regulatory mechanism in mice was unverified so far. Therefore, STAT3 levels in the liver tissue samples from the mice in each experimental group were examined immunohistochemically. The analysis revealed an up-regulation of STAT3 expression in the CCl_4_-induced liver fibrosis tissue samples, while AHWE assisted in reducing this undesirable trend (Figure 7A). Next, the protein levels of STAT3 and p-STAT3 in the liver samples were analyzed using immunoblotting, and the results are presented in Figure 7B. As visible in the figure, the p-STAT3 to STAT3 ratio was higher in the CCl_4_-induced HF mice liver tissue relative to the control, while the ratio was decreased upon treatment with AHWE. Furthermore, the expression levels of the *Stat3* gene in the liver samples were determined. A higher expression of STAT3 was detected in the HF tissues compared to the normal liver tissues. In the presence of AHWE, the mRNA expression levels of this gene decreased significantly in a dose-dependent manner (Figure 7C). These results provided evidence in support of the involvement of STAT3 signaling pathway in the *in vivo* suppression of HF upon AHWE treatment. Therefore, it was concluded that AHWE could restrain the activation of HSCs through the suppression of the STAT3 signaling pathway, which ultimately mitigated liver fibrosis.

## 4 Discussion

HF is characterized by the excessive accumulation of the extracellular matrix or the presence of excessive connective tissue in response to liver inflammation and chronic liver injury (Wynn and Ramalingam, 2012). As HF progresses, it often develops into irreversible cirrhosis or even liver cancer in certain cases, thereby raising a major threat to public health. Activated HSCs, which are capable of transforming into myofibroblast-like cells, are recognized as the effector cells in liver fibrosis (da Silva Meirelles et al., 2020). Therefore, HSCs are considered the main fibrogenic precursor cells in the liver. This activation of HSCs is characterized by the gradual release of intracellular vitamin A, an increased rate of cell proliferation, α-SMA activation, the synthesis of a fibrogenic matrix rich in Col Ⅰ and Col Ⅲ, and a higher expression of the extracellular matrix that distorts the liver architecture by forming liver fiber nodules and consequently disrupts the normal functioning of the liver, ultimately causing pathophysiological damage to the organ (Ding et al., 2021). The blockade and/or reversal of this activation of HSCs determines the efficacy of any anti-fibrotic therapy and is, therefore, the key target in HF treatment. The release of pro-inflammatory cytokines from hepatocytes and Kupffer cells is reported as a critical step in the pathogenesis of HF (Wu et al., 2019). The resulting activation of immune cells, such as monocytes, neutrophils, and lymphocytes, is also important for the development of liver injury (Adams et al., 2010). In the liver, *Tnf-α*, *Il6*, and *Il1β* may trigger the accumulation of neutrophils and monocytes, thereby contributing to the early stages of disease progression and maintaining persistent inflammation of the liver tissue (Kany et al., 2019).

The mechanisms underlying fibrosis are not completely understood to date, due to which no highly effective therapy is currently available for liver fibrosis. Therefore, it is imperative to explore and develop novel therapies and medicines for treating liver fibrosis. As botanical drugs are gaining increasing attention as sources of novel bioactive substances and have begun replacing synthetic pharmaceuticals, using herbal medicinal in HF treatment appears to be an attractive option as a viable and effective alternative strategy (Latief and Ahmad, 2018). *Amydrium hainanense* (H.Li, Y.Shiao & S.L.Tseng) H.Li is reported to have potential medicinal value in regard to liver diseases, especially liver inflammation. This unique Chinese climbing Fujimoto has been used widely as a folk medicine in the Yao region. A pre-experiment conducted by our research group also confirmed that the water extract of AH could exhibit anti-fibrogenic effects in mice with liver injury.

In this context, the present study pioneered in confirming the therapeutic value of AHWE in HF, both *in vivo* and *in vitro*, in addition to elucidating that this effect of AHWE was mainly based on the suppression of HSC activation and proliferation via STAT3 signaling pathway inhibition. The liver fibrosis model was established using 20% CCl_4_. The hepatic pathology analysis based on H&E staining confirmed the successful establishment of typical liver fibrosis in the model group mice. Upon AHWE treatment, improvement in the hepatic lobule structure and reduced inflammatory cell infiltration was observed in a dose-dependent manner. The ALT and AST levels are low under physiological conditions in normal hepatocytes, while large amounts of these enzymes are released into the blood when the hepatocytes are damaged, which results in a sharp increase in the serum activities of ALT and AST (Kim et al., 2020). Therefore, serum ALT and AST levels are two typical and major biochemical parameters marking liver injury. In the present study, the serum activities of ALT and AST exhibited an upward trend in the model group, indicating declining liver function. This undesirable pathological damage to the liver tissue was improved upon AHWE treatment. The liver sections stained using Masson’s trichrome stain exhibited hepatic histopathological changes, including damaged lobular structure and excessive fat and collagen deposition in the model mice, and these changes were significantly improved upon AHWE treatment. The various examinations of different factors associated with liver fibrosis revealed that, compared to the CCl_4_ group, the AHWE administration group exhibited decreased levels of Col Ⅰ, hydroxyproline, and α-SMA protein. Among the well-recognized mediators, TNF-α, IL-6, and Il1β have been implicated in the regulation of inflammation in liver diseases. TNF-α reportedly induces hepatocyte proliferation, apoptosis, and inflammation, in addition to suppressing Col Ⅰ gene expression (Yang and Seki, 2015). IL-6 has reportedly presented the strongest correlation with fibrosis severity in biopsy patients (Ridner et al., 2018). The q-PCR analysis results of the present study revealed that the expression levels of *Acta2*, *Col1a1*, *Col3a1*, *Tnfa*, *Il6*, and *Il1β* in the liver tissue samples were decreased upon treatment with AHWE. These data suggested that AHWE exerted an anti-fibrotic effect on the CCl4-induced HF mice. Inspired by the results of *in vivo* experiments, the anti-fibrosis effects of AHWE and the underlying mechanisms were studied *in vitro* as well.

The HSC-T6 cell line is a valuable cell model for studies of retinoid metabolism based on their similar retinoid phenotype as primary cell, which makes it an important tool for studying liver fibrosis *in vitro*. In addition, RAW264.7 is considered to be one of the best models of macrophages. These two different cell types could provide a guarantee for phenotypic verification and mechanism study. TGF-β1 was used for directly stimulating the HSC-T6 cells to establish the *in vitro* liver fibrosis model, while LPS was employed to induce the RAW264.7 cells to establish the *in vitro* inflammation model as these are currently the relatively mature experimental modeling methods available for the cellular level (Dewidar et al., 2019) (Zhou et al., 2021). The potential cytotoxicity of AHWE toward these two types of cells was assessed using the CCK-8 assay kit. In the drug concentration range that had almost no effect on the cell viability in both cells, three appropriate drug concentrations were selected for evaluation in subsequent experiments. Consistent with the *in vivo* results, the immunohistochemical examination revealed that AHWE suppressed α-SMA expression. The changes in the expressions of the genes associated with fibrosis and inflammation, including *Acta2*, *Col1a1*, *Col3a1*, *Tnfa*, *Il6*, and *Il1β*, were also significantly reversed upon AHWE treatment. It was observed that AHWE could inhibit the activation of HSCs and the inflammatory process. Among the various downstream signaling targets of HF, STAT and its family members, particularly STAT1 and STAT3 proteins, play an important role in the pathogenesis of HF. STAT3 is a transcription factor associated with the proliferation and activation of HSCs, and could, therefore, contribute to HF (Xu et al., 2014). In response to the cytokines and growth factors released upon liver injury, Janus tyrosine kinases phosphorylate STAT3, following which it dimerizes and translocates to the nucleus, where it plays its role involving the transcriptional regulation of its target genes (Jatiani et al., 2010). The results of the present study indicated that LPS stimulation increased the p-STAT3 to STAT3 ratio relative to the control group in RAW264.7 cells. In addition, AHWE treatment decreased the protein expression levels of p-STAT3/STAT3 and the mRNA expressions of STAT3. Therefore, it was speculated that the inhibitory effect of AHWE on liver fibrosis was related to the activation of the STAT3 signaling pathway.

In the next experiment, STAT3 was overexpressed in the HSC-T6 and RAW264.7 cell lines using the pEGFP-STAT3 plasmid. Western blotting was performed to detect the STAT3 protein bands. The STAT3 protein levels were observed to be significantly higher in the plasmid transfected cells compared to the cells transfected with the empty vector. These results were validated in an independent cohort of samples using quantitative PCR. Therefore, it was inferred that the STAT3 overexpression cell line was successfully established. The expression levels of α-SMA protein, as revealed in the western blot analysis, validated that AHWE exerted an inhibitory effect on the TGF-β1stimulation-induced elevated SMA levels. However, the levels of the fibrosis marker α-SMA were significantly increased upon STAT3 overexpression, suggesting that STAT3 mitigated liver fibrosis. Furthermore, it was observed that the levels of *Acta2*, *Col1a1*, *Col3a1*, *Tnfa*, *Il6*, and *Il1β* genes were significantly increased upon STAT3 overexpression, again suggesting that STAT3 mitigated liver fibrosis. These results further elucidated the mediating role of the STAT3 signaling pathway in the anti-fibrotic effect of AHWE *in vitro*. In order to verify the regulatory mechanism underlying this effect in mice that was consistent with the results of *in vitro* experiments, the expression levels of STAT3 in the liver tissue samples were determined using the immunohistochemical examination. The results presented in Figure 7A confirmed the upregulation of STAT3 expression in CCl_4_-induced liver fibrosis tissue samples (tan-colored staining). This adverse effect was observed to decrease upon AHWE treatment. Moreover, the p-STAT3 to STAT3 ratio was increased in the CCl_4_-induced HF mice, while the ratio was decreased upon AHWE treatment. The expression levels of the *Stat3* gene were determined in the liver samples. It was observed that *STAT3* expression was higher in the HF tissues than that in the normal liver tissues. In the presence of AHWE, the mRNA expression levels of this gene were decreased evidently in a dose-dependent manner (Figure 7C). The above results provided evidence that the regulation of the STAT3 signaling pathway was involved in the *in vivo* suppression of HF upon AHWE treatment.

## 5 Conclusion

The present study pioneered in demonstrating the potential anti-HF effects of AHWE. Moreover, it was demonstrated these effects of AHWE were, at least in part, due to the inhibition of STAT3 signaling. The findings of the present study would serve as a strong pharmacological basis for designing a target-based therapy against HF, as well as for the utilization of AHWE as a novel phytotherapeutic agent in HF treatment.

## Data Availability

The original contributions presented in the study are included in the article/Supplementary Material, further inquiries can be directed to the corresponding authors.

## Glossary

Aps: ammonium persulfate
Acta2: actin alpha 2
AH: *Amydrium hainanense* (H.Li, Y.Shiao & S.L.Tseng) H.Li
AHWE: water extract of *Amydrium hainanense* (H.Li, Y.Shiao & S.L.Tseng) H.Li
ALT: alanine transaminase
α-SMA: alpha smooth muscle actin
AST: aspartate transaminase
BCA: bicinchoninic acid assay
CCl_4_: Carbon tetrachloride
Col Ⅰ: collagen 1
Col Ⅲ: collagen 3
Col1a1: collagen 1a1
Col3a1: collagen 3a1
DAB: Diaminobenzidine tertrahydrochloride
DMEM: Dulbecco’s modified eagle medium
DMSO: Dimethyl sulfoxide
ECL: enhanced chemical luminescence
EDTA: ethylenediaminetetraacetic acid
ECM: extracellular matrix
FBS: Fetal bovine serum
GAPDH: glyceraldehyde-3-phosphate dehydrogenase
H&E: hematoxylin and eosin
HF: hepatic fibrosis
HSCs: hepatic stellate cells
LPS: lipopolysaccharide
IL-1β: interleukin-1 beta
IL-6: interleukin-6
PAGE: polyacrylamide gel electrophoresis
PBS: phosphate buffer Saline
PVDF: polyvinylidene fiuoride
SDS: sodium dodecyl sulfate
STAT3: signal transducers and activators of transcription 3
TBS: Tris buffered saline
TEMED: N, N, N′, N′-Tetramethylethylenediamine
Tris: Tris (hydroxymethyl) aminomethane
TNF-α: tumor necrosis factor-α
TGF-β1: transforming growth factor beta 1
