# Supplementary material for: Yao medicine Amydrium hainanense suppresses hepatic fibrosis by repressing hepatic stellate cell activation via STAT3 signaling
Source: Front Pharmacol. 2022 Dec 14;13:1043022. doi: 10.3389/fphar.2022.1043022 (PMC9794994; doi:10.3389/fphar.2022.1043022)
Supplement: Supplementary file 1 [file Presentation1.PPTX]

## Slide 1
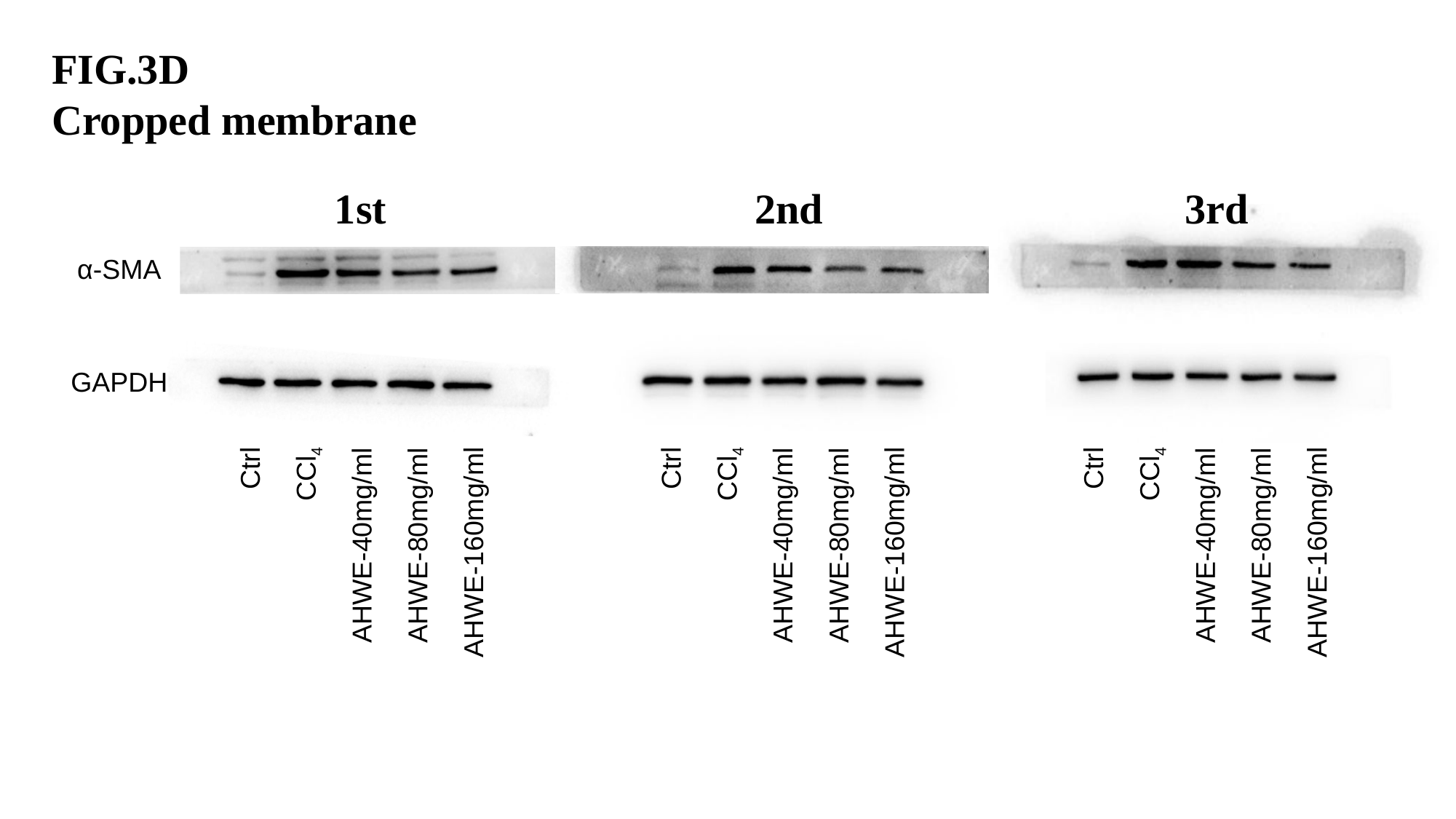

FIG.3D
Cropped membrane
1st
2nd
3rd
α-SMA
GAPDH
Ctrl
CCl4
AHWE-40mg/ml
Ctrl
CCl4
AHWE-40mg/ml
AHWE-80mg/ml
AHWE-80mg/ml
Ctrl
CCl4
AHWE-40mg/ml
AHWE-80mg/ml
AHWE-160mg/ml
AHWE-160mg/ml
AHWE-160mg/ml

## Slide 2
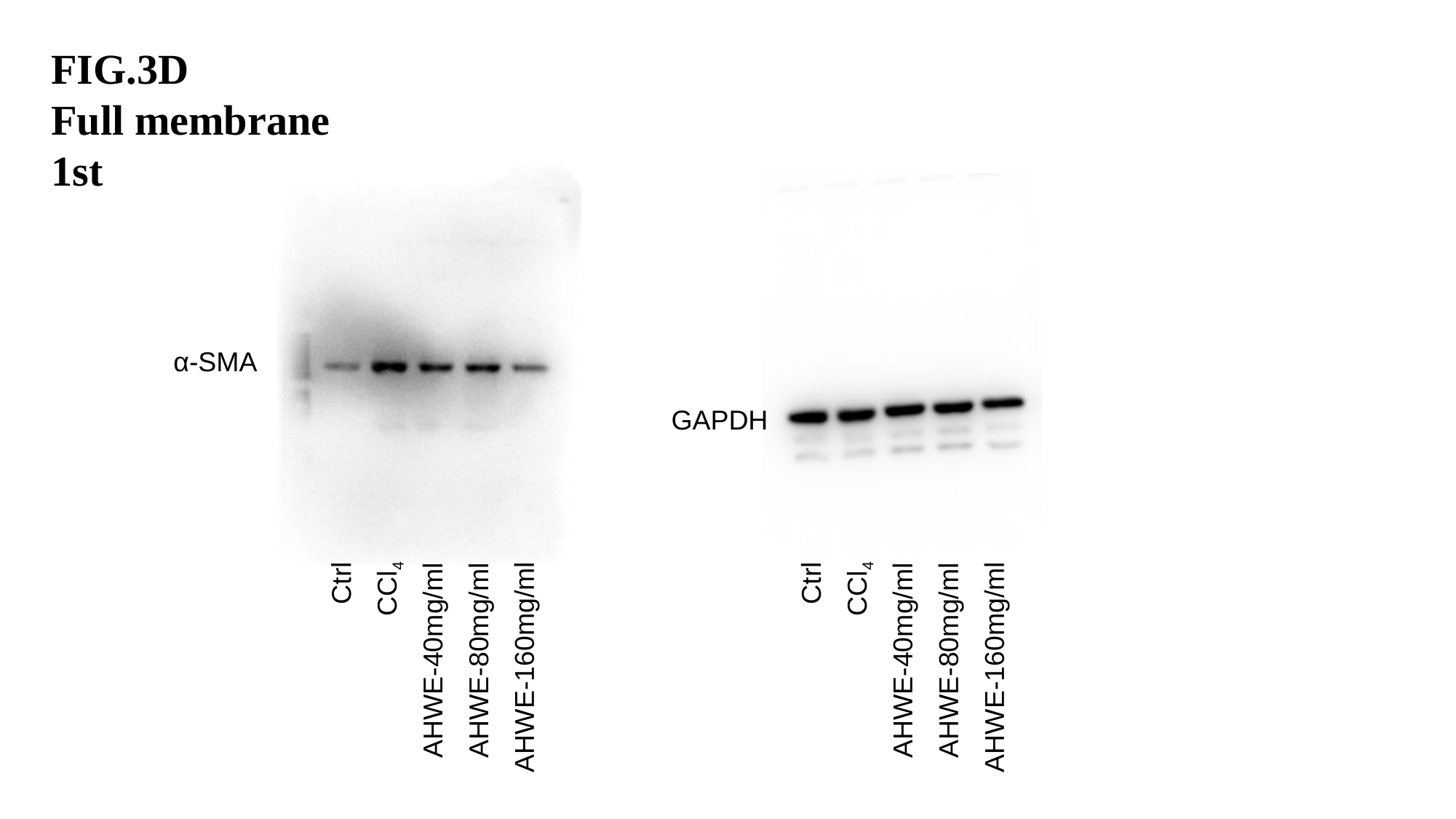

FIG.3D
Full membrane
1st
α-SMA
GAPDH
Ctrl
CCl4
AHWE-40mg/ml
AHWE-80mg/ml
AHWE-160mg/ml
Ctrl
CCl4
AHWE-40mg/ml
AHWE-80mg/ml
AHWE-160mg/ml

## Slide 3
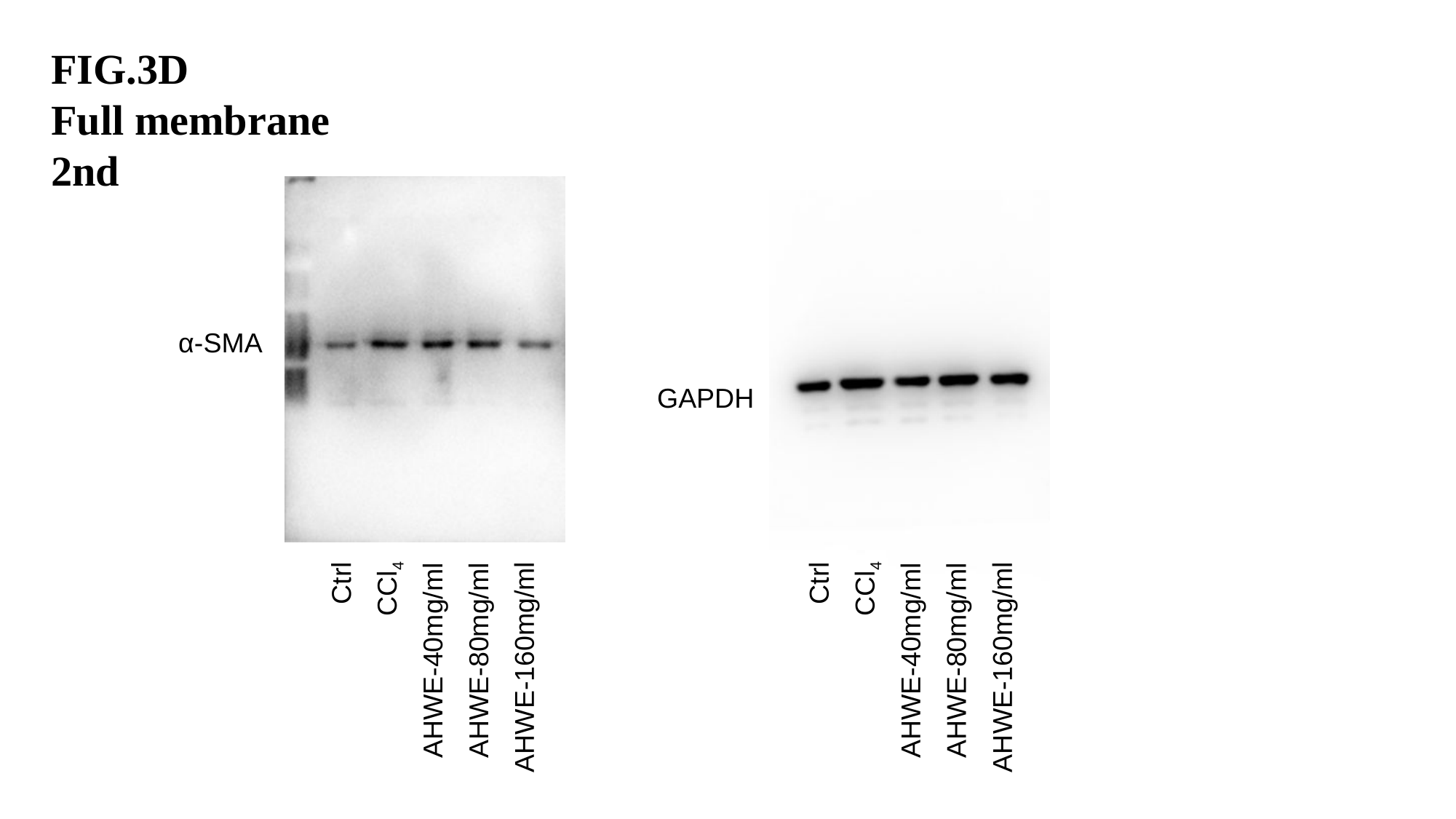

FIG.3D
Full membrane
2nd
α-SMA
GAPDH
Ctrl
CCl4
AHWE-40mg/ml
AHWE-80mg/ml
AHWE-160mg/ml
Ctrl
CCl4
AHWE-40mg/ml
AHWE-80mg/ml
AHWE-160mg/ml

## Slide 4
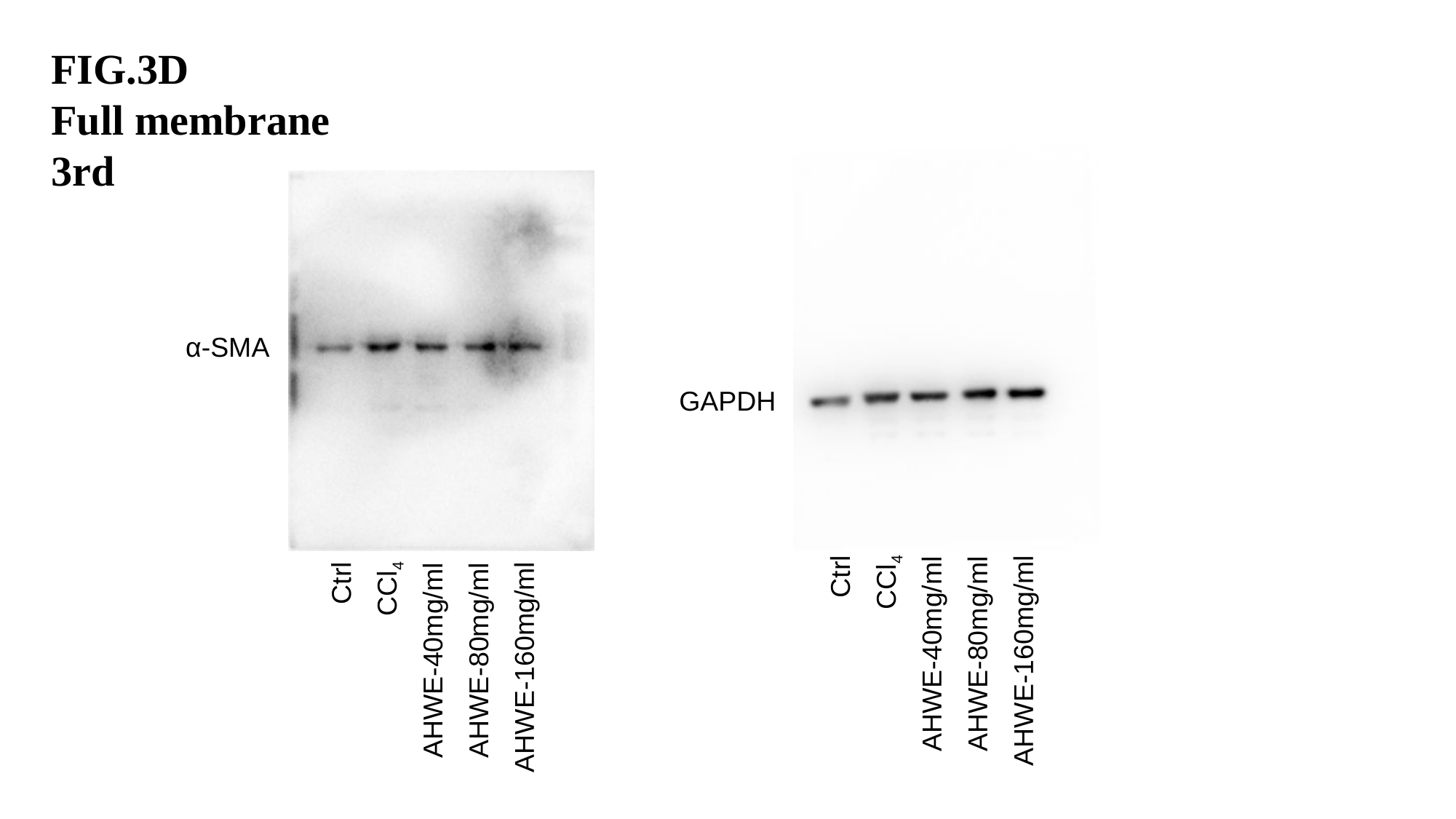

FIG.3D
Full membrane
3rd
α-SMA
GAPDH
Ctrl
CCl4
AHWE-40mg/ml
AHWE-80mg/ml
AHWE-160mg/ml
Ctrl
CCl4
AHWE-40mg/ml
AHWE-80mg/ml
AHWE-160mg/ml

## Slide 5
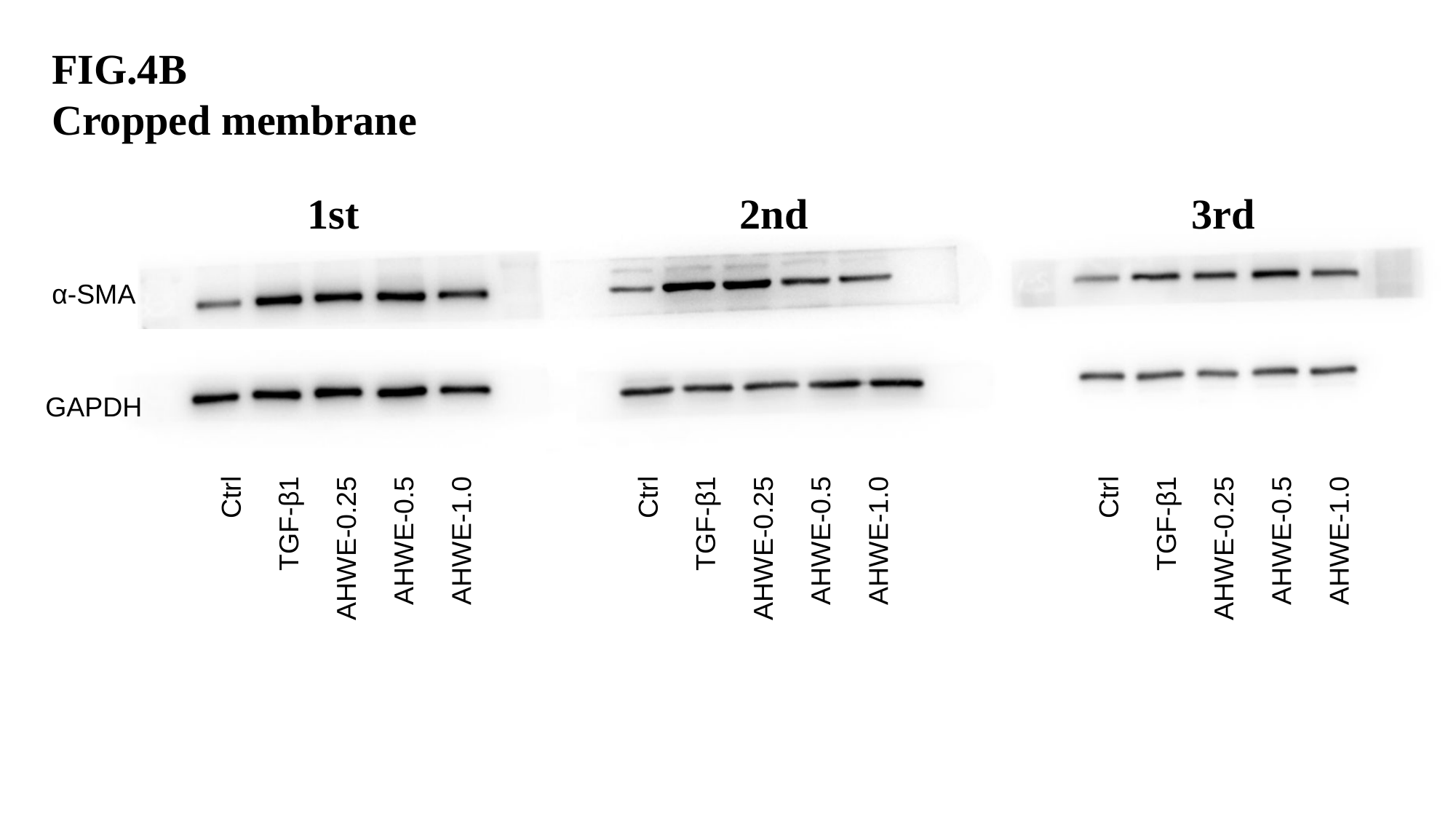

FIG.4B
Cropped membrane
1st
2nd
3rd
α-SMA
GAPDH
Ctrl
TGF-β1
AHWE-0.25
AHWE-0.5
Ctrl
TGF-β1
AHWE-0.25
AHWE-0.5
Ctrl
TGF-β1
AHWE-0.25
AHWE-0.5
AHWE-1.0
AHWE-1.0
AHWE-1.0

## Slide 6
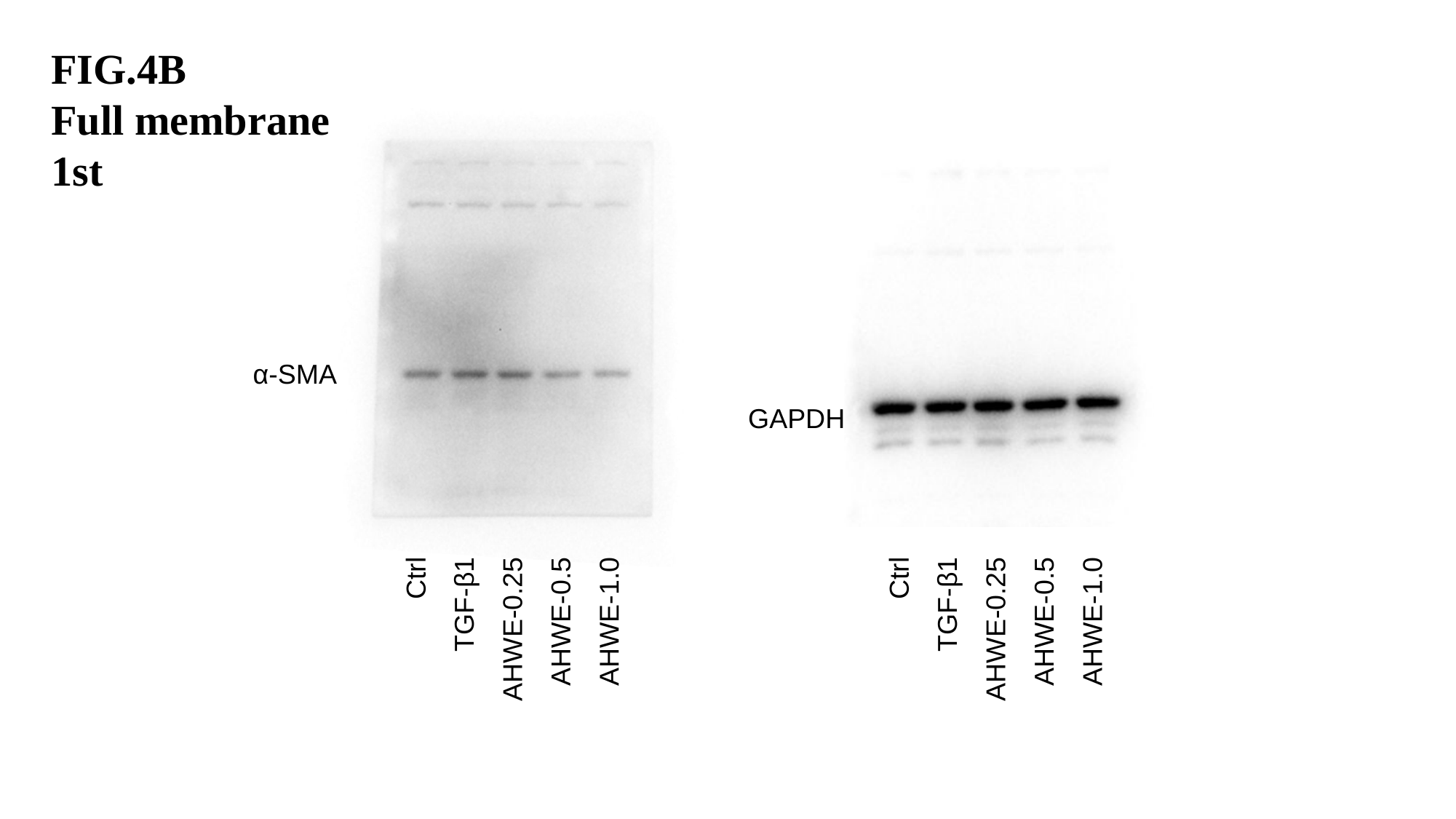

FIG.4B
Full membrane
1st
α-SMA
GAPDH
Ctrl
TGF-β1
AHWE-0.25
AHWE-0.5
AHWE-1.0
Ctrl
TGF-β1
AHWE-0.25
AHWE-0.5
AHWE-1.0

## Slide 7
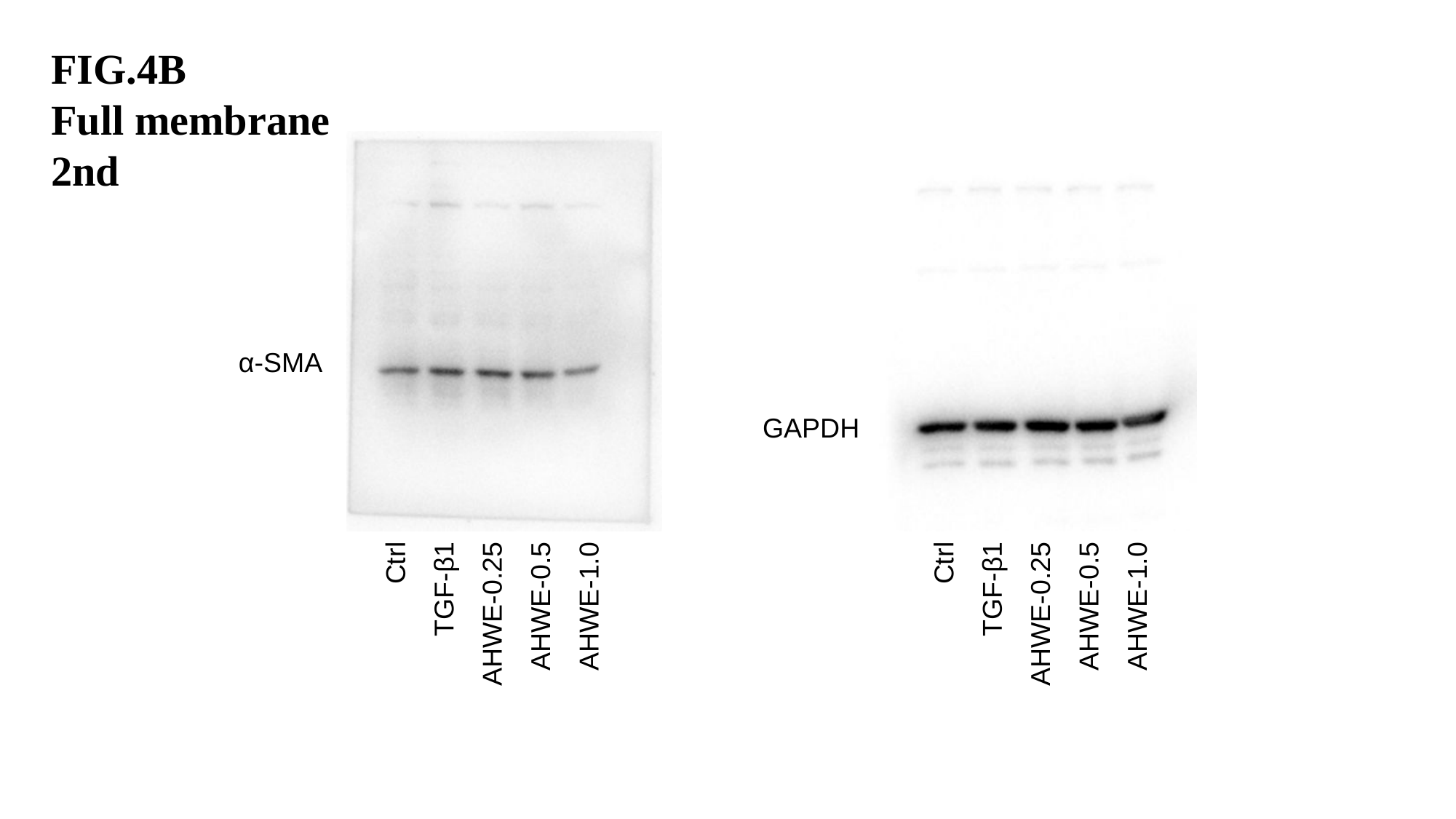

FIG.4B
Full membrane
2nd
α-SMA
GAPDH
Ctrl
TGF-β1
AHWE-0.25
AHWE-0.5
AHWE-1.0
Ctrl
TGF-β1
AHWE-0.25
AHWE-0.5
AHWE-1.0

## Slide 8
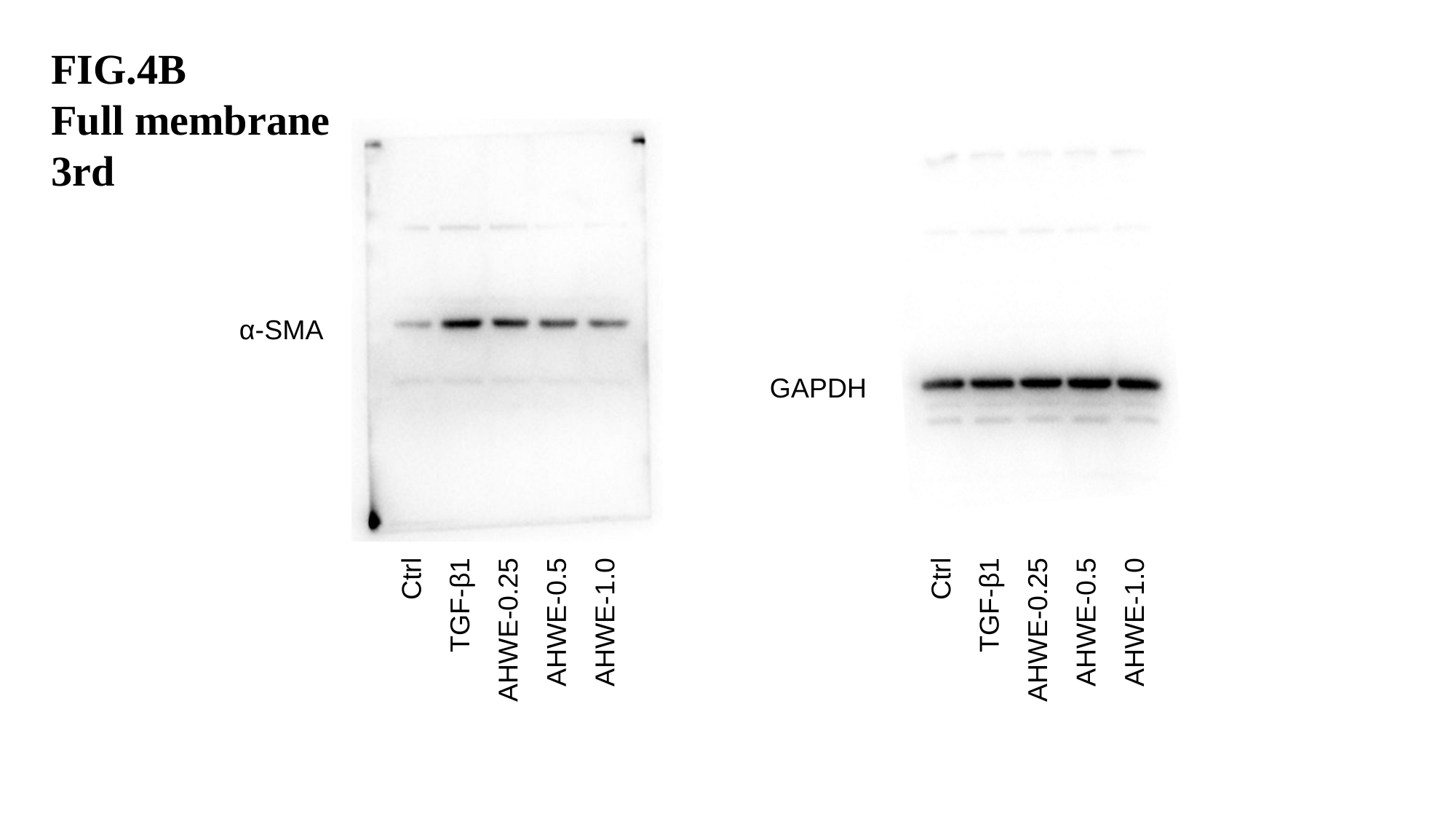

FIG.4B
Full membrane
3rd
α-SMA
GAPDH
Ctrl
TGF-β1
AHWE-0.25
AHWE-0.5
AHWE-1.0
Ctrl
TGF-β1
AHWE-0.25
AHWE-0.5
AHWE-1.0

## Slide 9
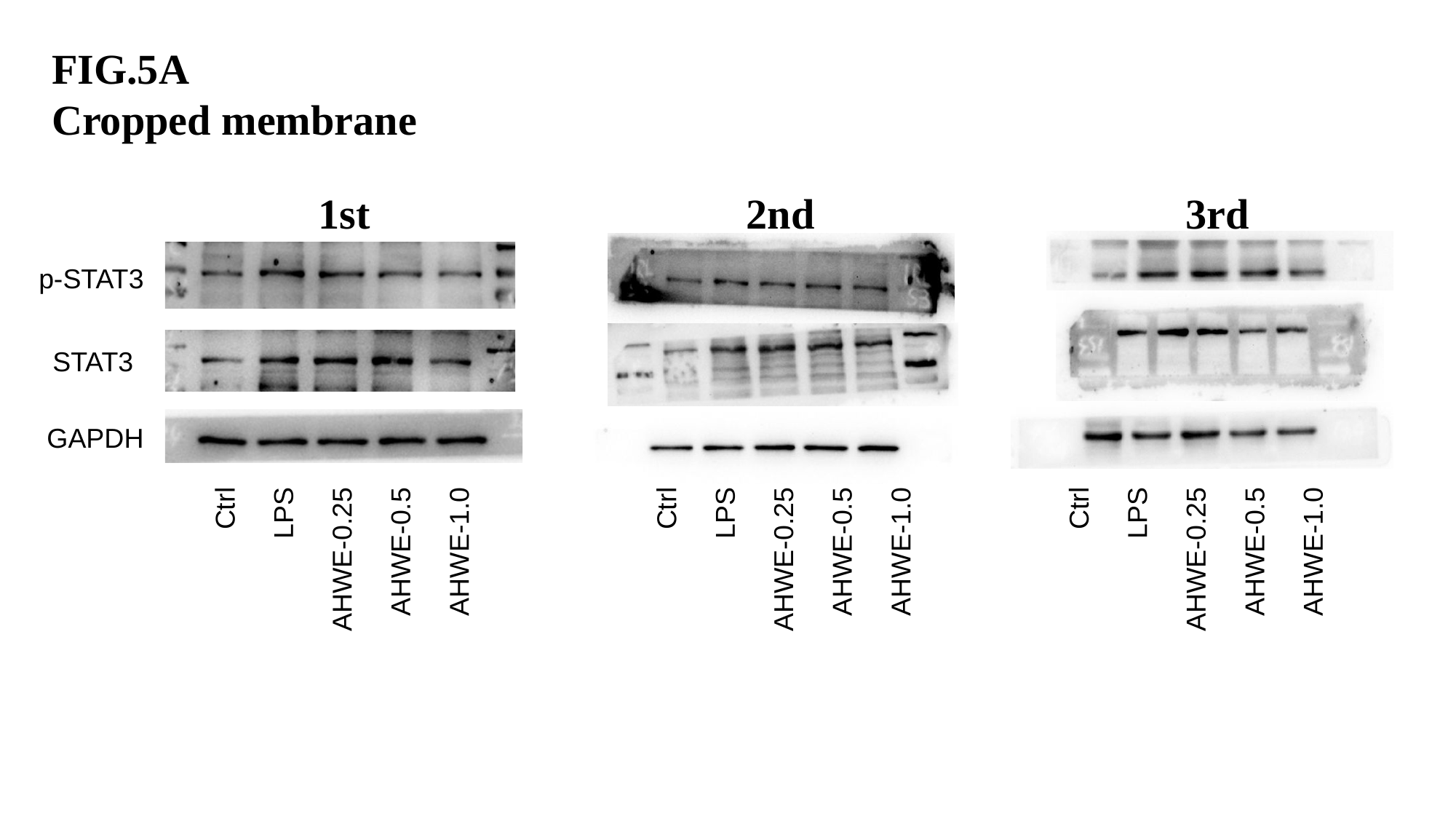

FIG.5A
Cropped membrane
1st
2nd
3rd
p-STAT3
STAT3
GAPDH
Ctrl
LPS
AHWE-0.25
AHWE-0.5
Ctrl
LPS
AHWE-0.25
AHWE-0.5
Ctrl
LPS
AHWE-0.25
AHWE-0.5
AHWE-1.0
AHWE-1.0
AHWE-1.0

## Slide 10
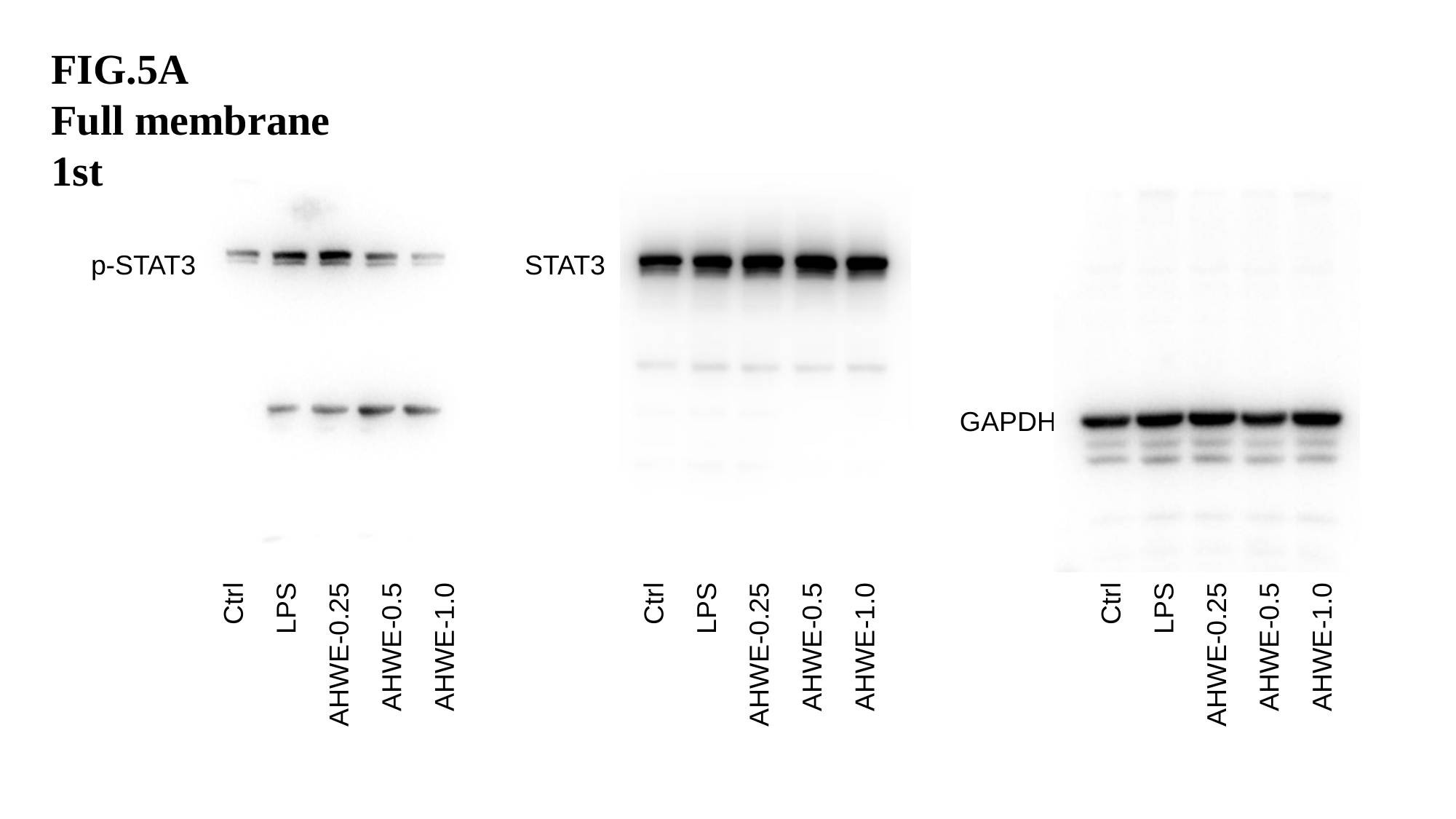

FIG.5A
Full membrane
1st
p-STAT3
STAT3
GAPDH
Ctrl
LPS
AHWE-0.25
AHWE-0.5
AHWE-1.0
Ctrl
LPS
AHWE-0.25
AHWE-0.5
AHWE-1.0
Ctrl
LPS
AHWE-0.25
AHWE-0.5
AHWE-1.0

## Slide 11
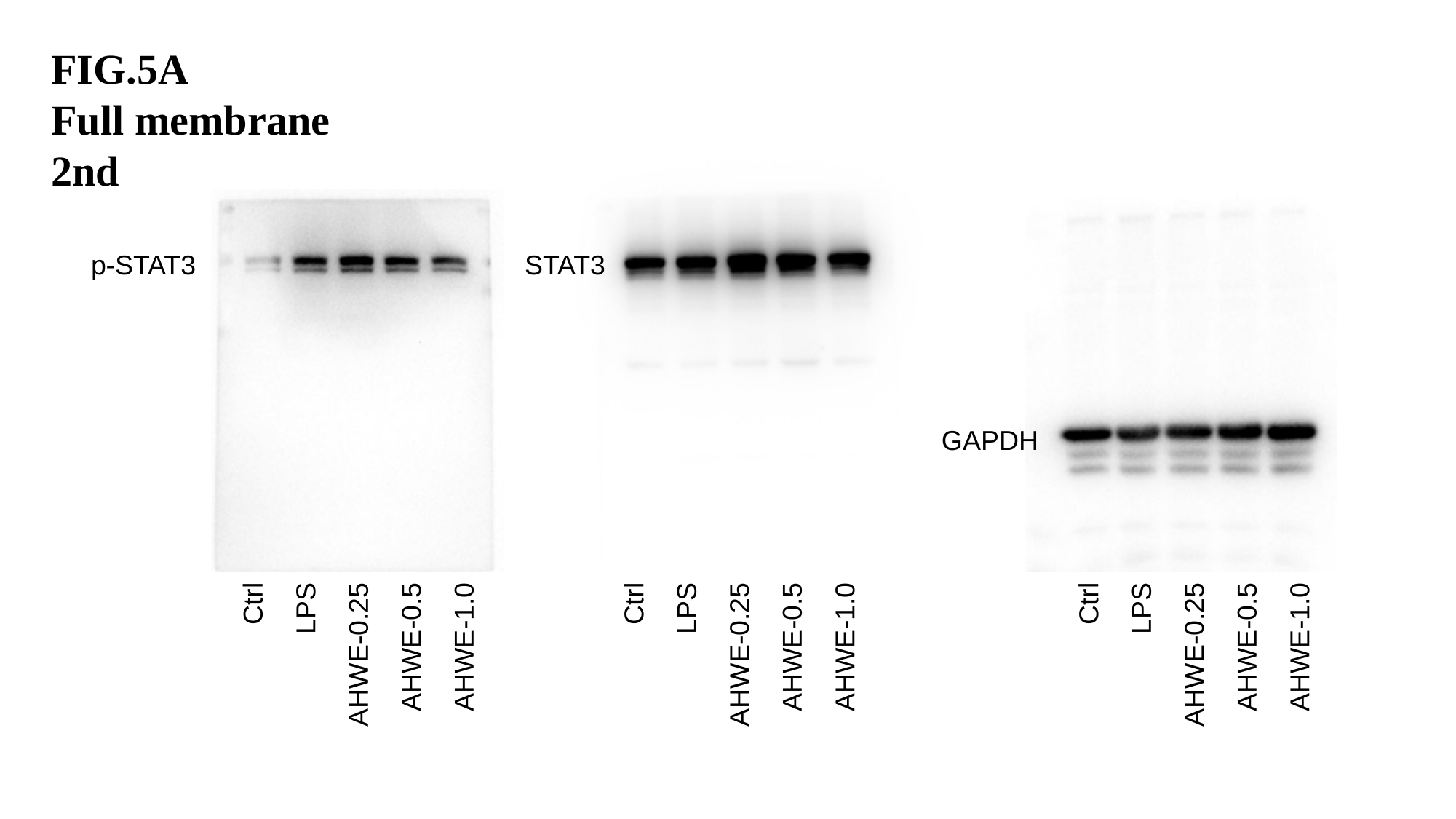

FIG.5A
Full membrane
2nd
p-STAT3
STAT3
GAPDH
Ctrl
LPS
AHWE-0.25
AHWE-0.5
AHWE-1.0
Ctrl
LPS
AHWE-0.25
AHWE-0.5
AHWE-1.0
Ctrl
LPS
AHWE-0.25
AHWE-0.5
AHWE-1.0

## Slide 12
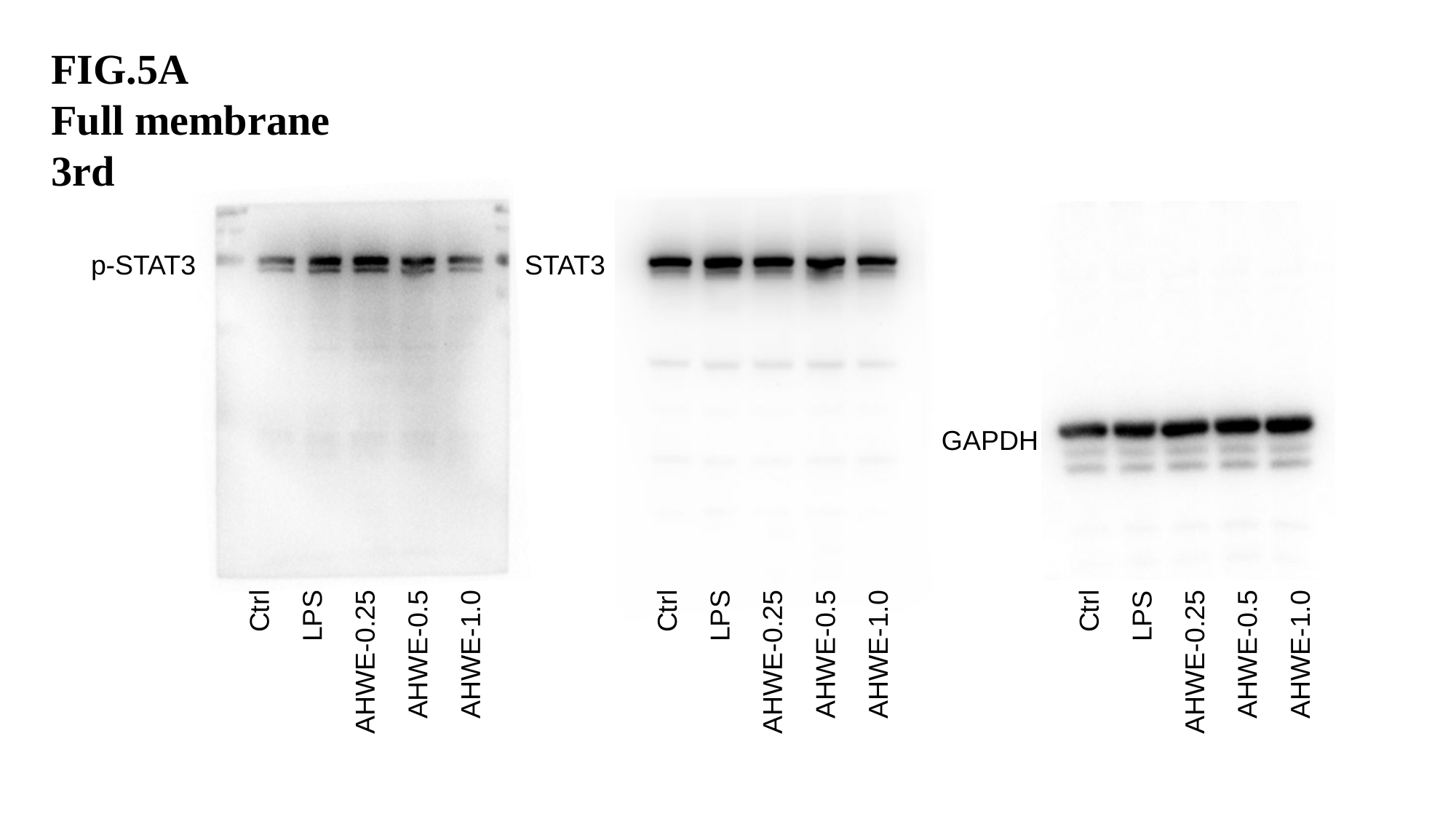

FIG.5A
Full membrane
3rd
p-STAT3
STAT3
GAPDH
Ctrl
LPS
AHWE-0.25
AHWE-0.5
AHWE-1.0
Ctrl
LPS
AHWE-0.25
AHWE-0.5
AHWE-1.0
Ctrl
LPS
AHWE-0.25
AHWE-0.5
AHWE-1.0

## Slide 13
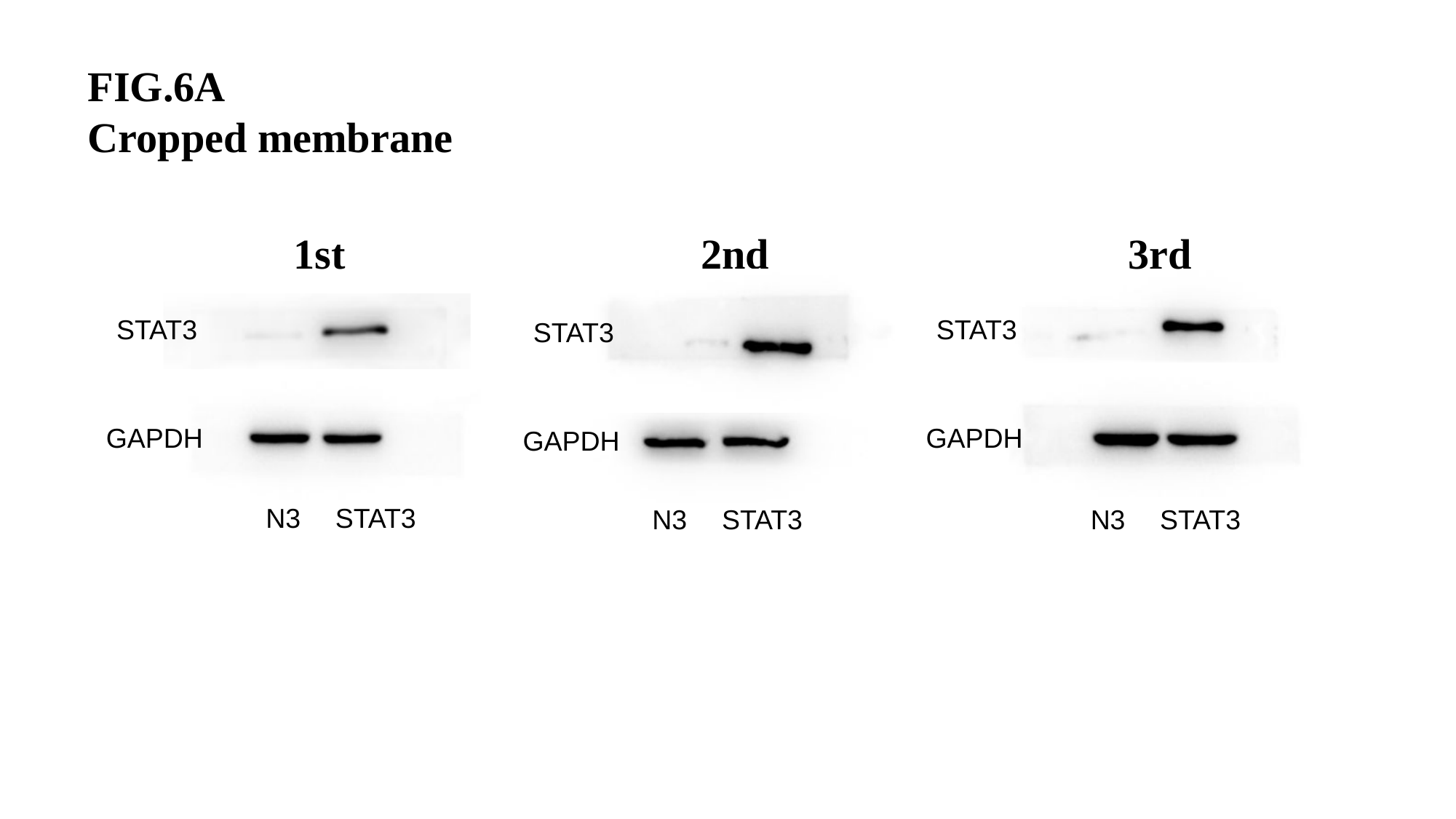

FIG.6A
Cropped membrane
1st
2nd
3rd
STAT3
GAPDH
STAT3
GAPDH
STAT3
GAPDH
N3
STAT3
N3
STAT3
N3
STAT3

## Slide 14
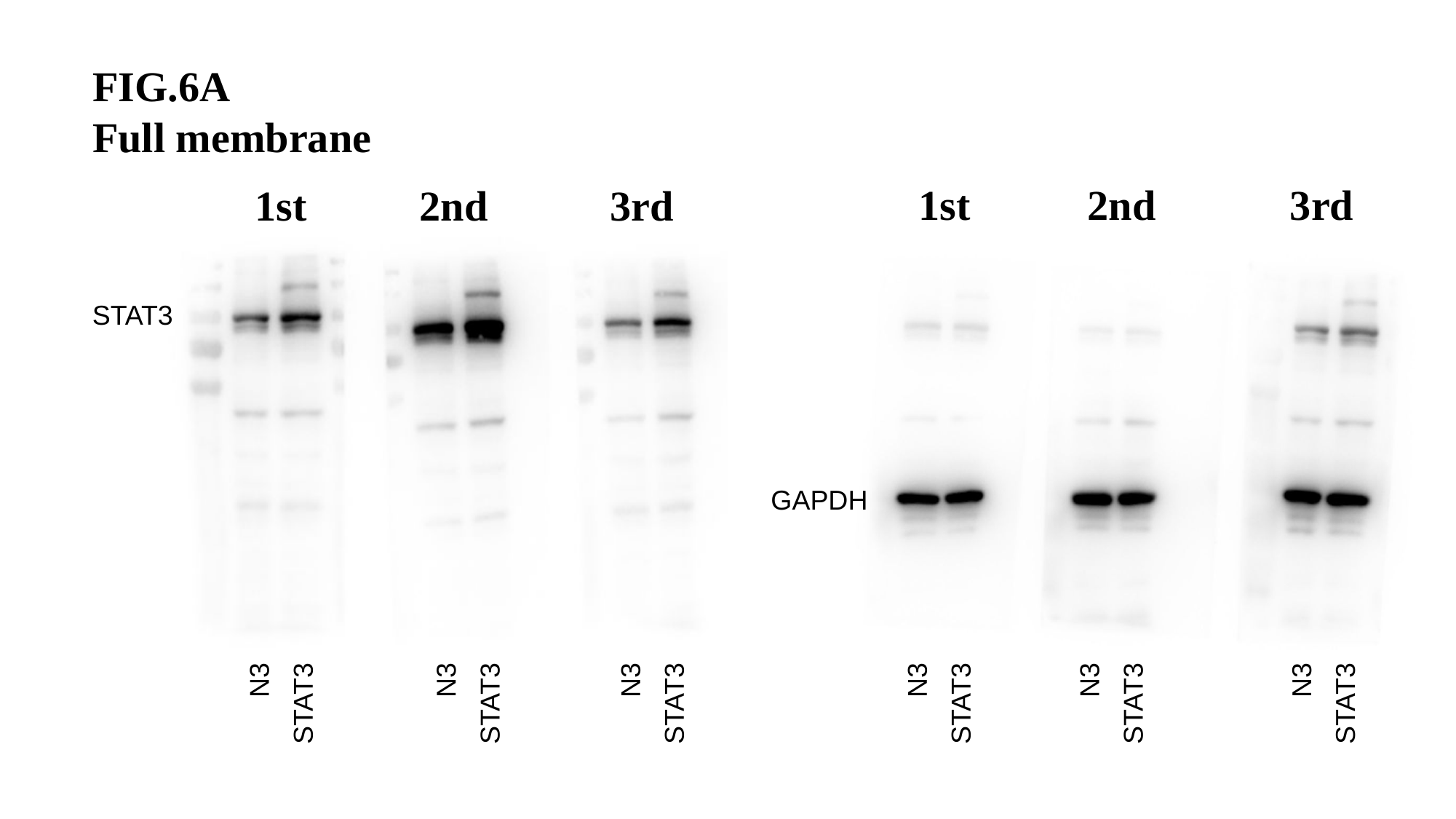

FIG.6A
Full membrane
1st
2nd
3rd
1st
2nd
3rd
STAT3
GAPDH
N3
STAT3
N3
STAT3
N3
STAT3
N3
STAT3
N3
STAT3
N3
STAT3

## Slide 15
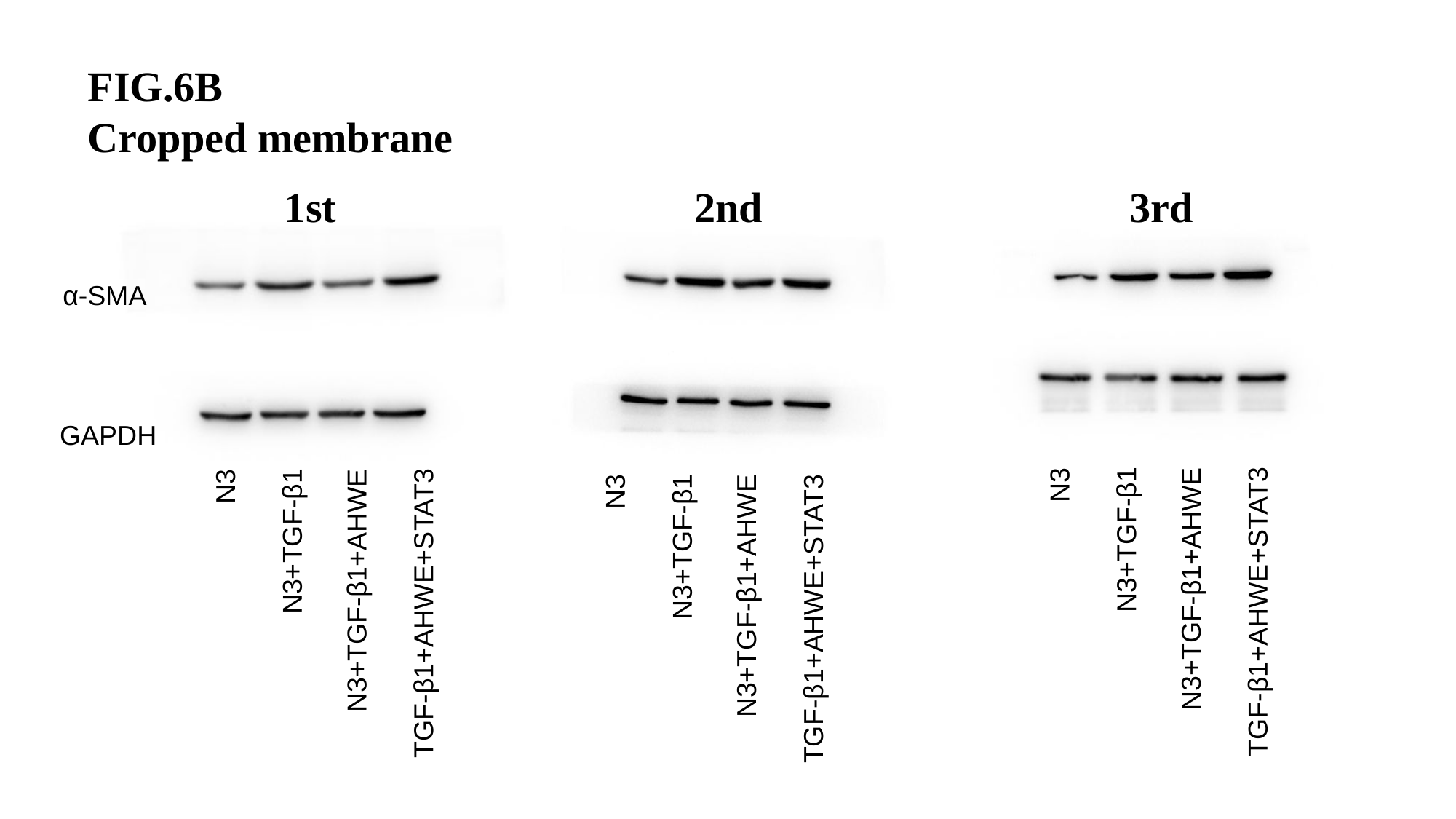

FIG.6B
Cropped membrane
1st
2nd
3rd
α-SMA
GAPDH
N3+TGF-β1
N3
N3+TGF-β1
N3
N3+TGF-β1
N3
N3+TGF-β1+AHWE
N3+TGF-β1+AHWE
N3+TGF-β1+AHWE
TGF-β1+AHWE+STAT3
TGF-β1+AHWE+STAT3
TGF-β1+AHWE+STAT3

## Slide 16
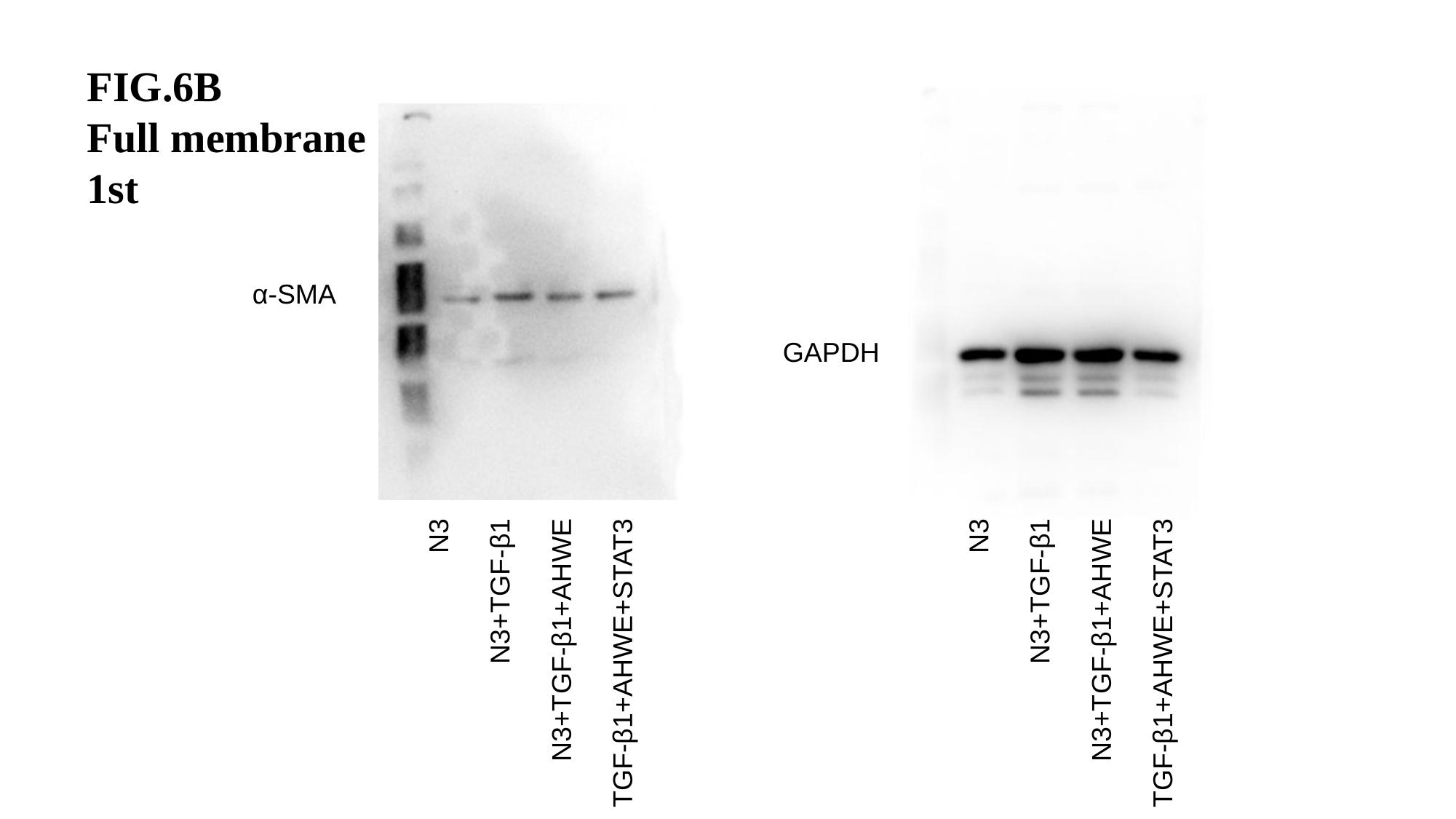

FIG.6B
Full membrane
1st
α-SMA
GAPDH
N3+TGF-β1
N3
N3+TGF-β1+AHWE
TGF-β1+AHWE+STAT3
N3+TGF-β1
N3
N3+TGF-β1+AHWE
TGF-β1+AHWE+STAT3

## Slide 17
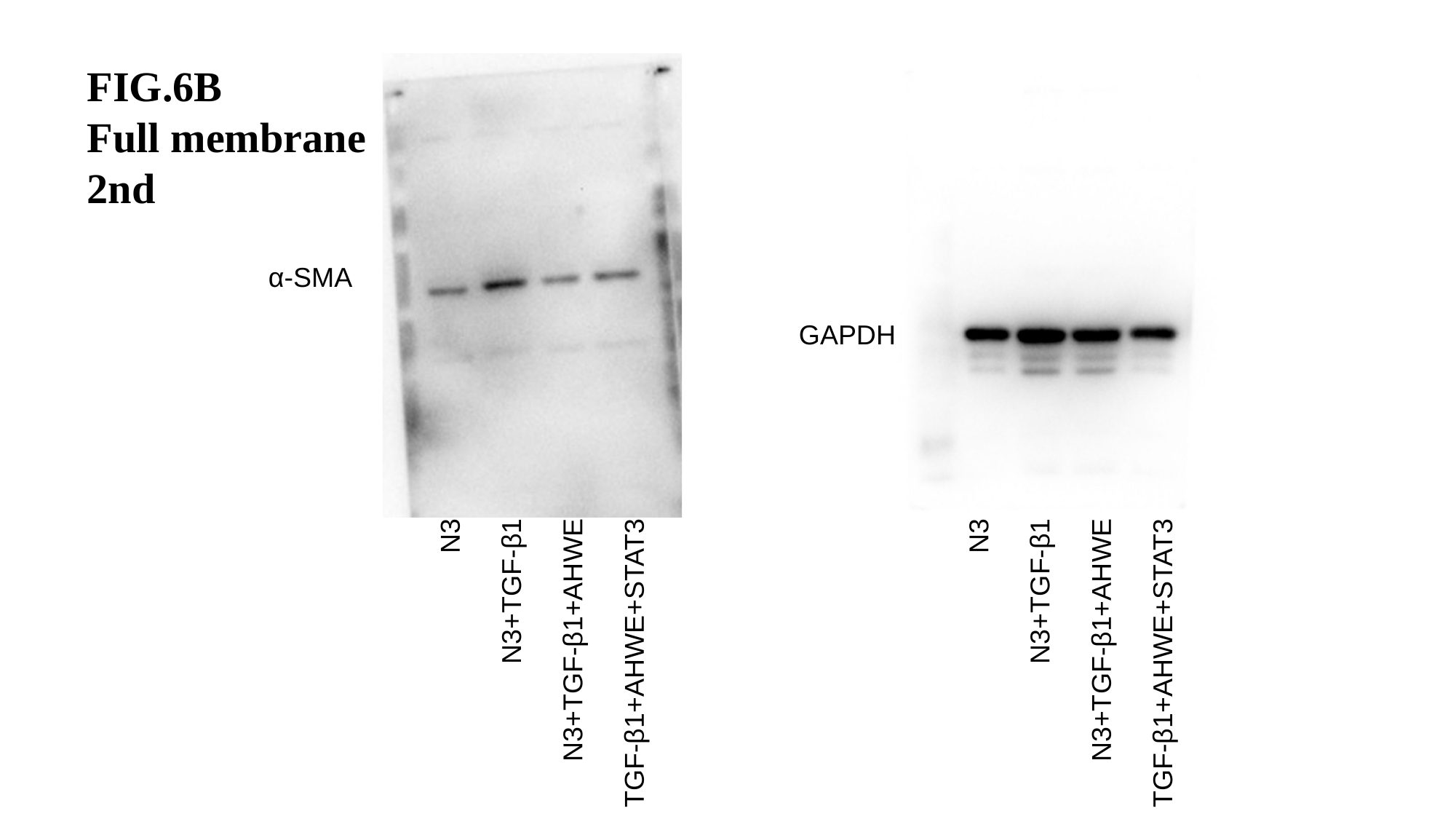

FIG.6B
Full membrane
2nd
α-SMA
GAPDH
N3+TGF-β1
N3
N3+TGF-β1+AHWE
TGF-β1+AHWE+STAT3
N3+TGF-β1
N3
N3+TGF-β1+AHWE
TGF-β1+AHWE+STAT3

## Slide 18
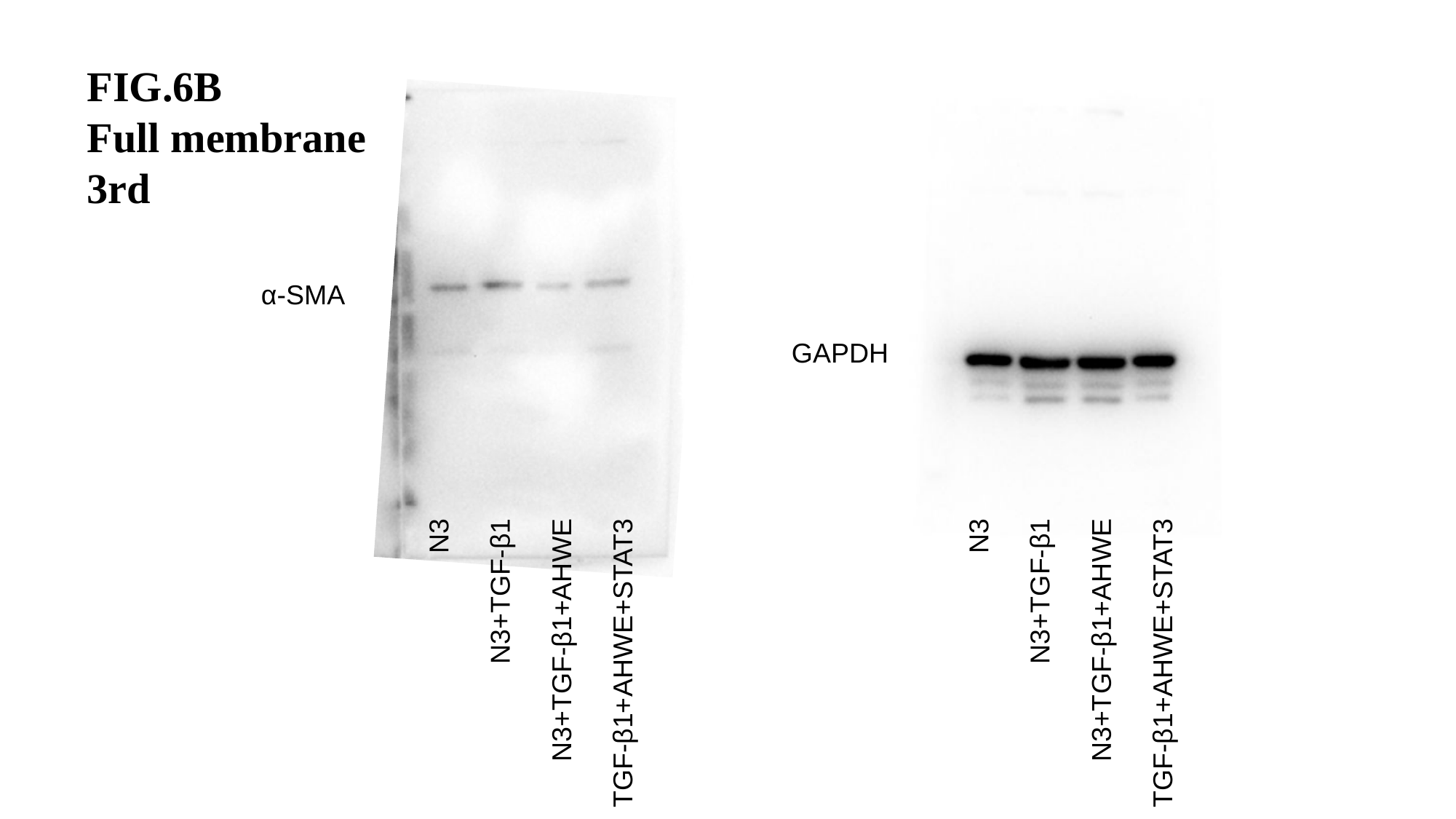

FIG.6B
Full membrane
3rd
α-SMA
GAPDH
N3+TGF-β1
N3
N3+TGF-β1+AHWE
TGF-β1+AHWE+STAT3
N3+TGF-β1
N3
N3+TGF-β1+AHWE
TGF-β1+AHWE+STAT3

## Slide 19
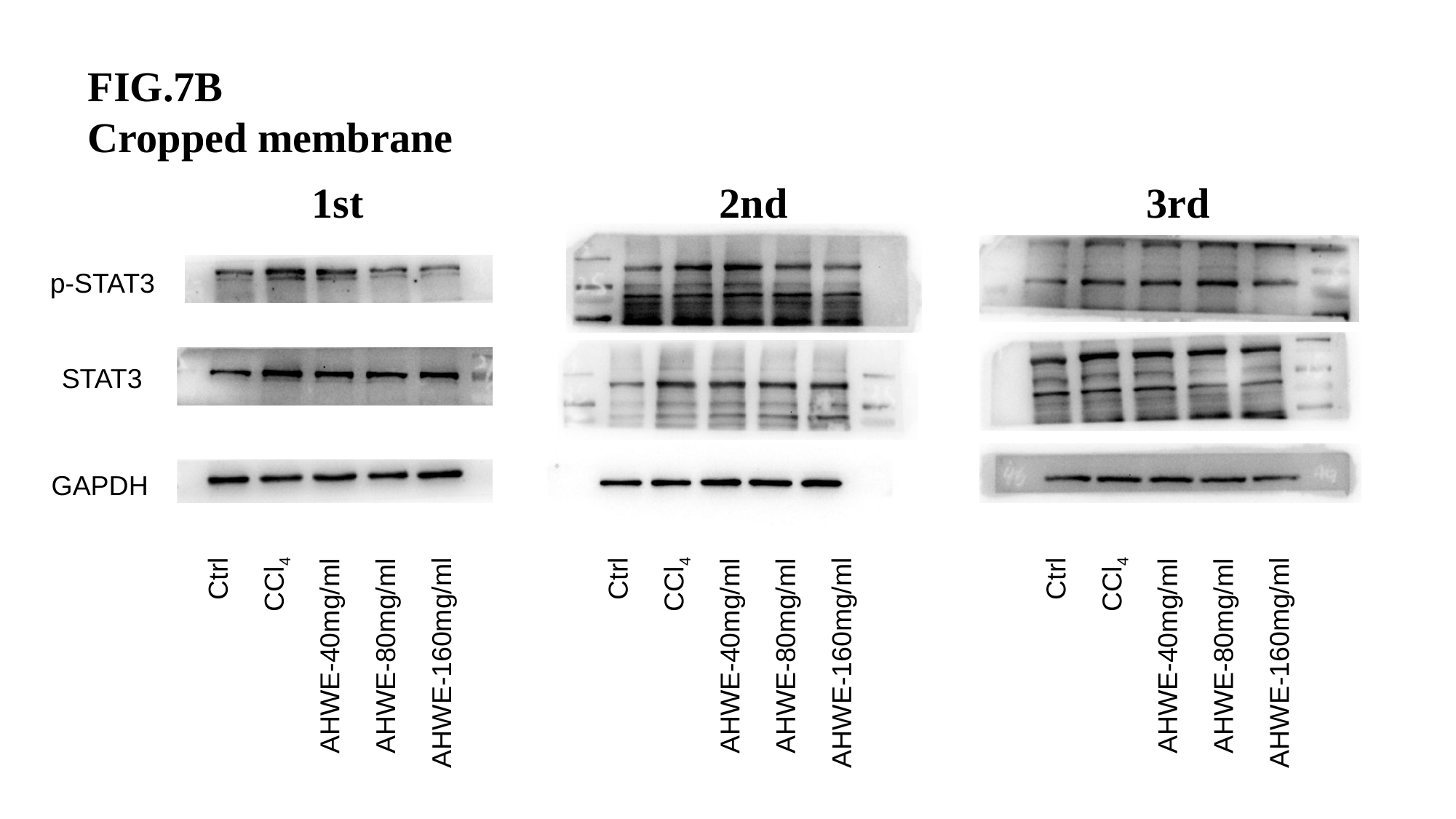

FIG.7B
Cropped membrane
1st
2nd
3rd
p-STAT3
STAT3
GAPDH
Ctrl
CCl4
AHWE-40mg/ml
Ctrl
CCl4
AHWE-40mg/ml
AHWE-80mg/ml
Ctrl
CCl4
AHWE-40mg/ml
AHWE-80mg/ml
AHWE-80mg/ml
AHWE-160mg/ml
AHWE-160mg/ml
AHWE-160mg/ml

## Slide 20
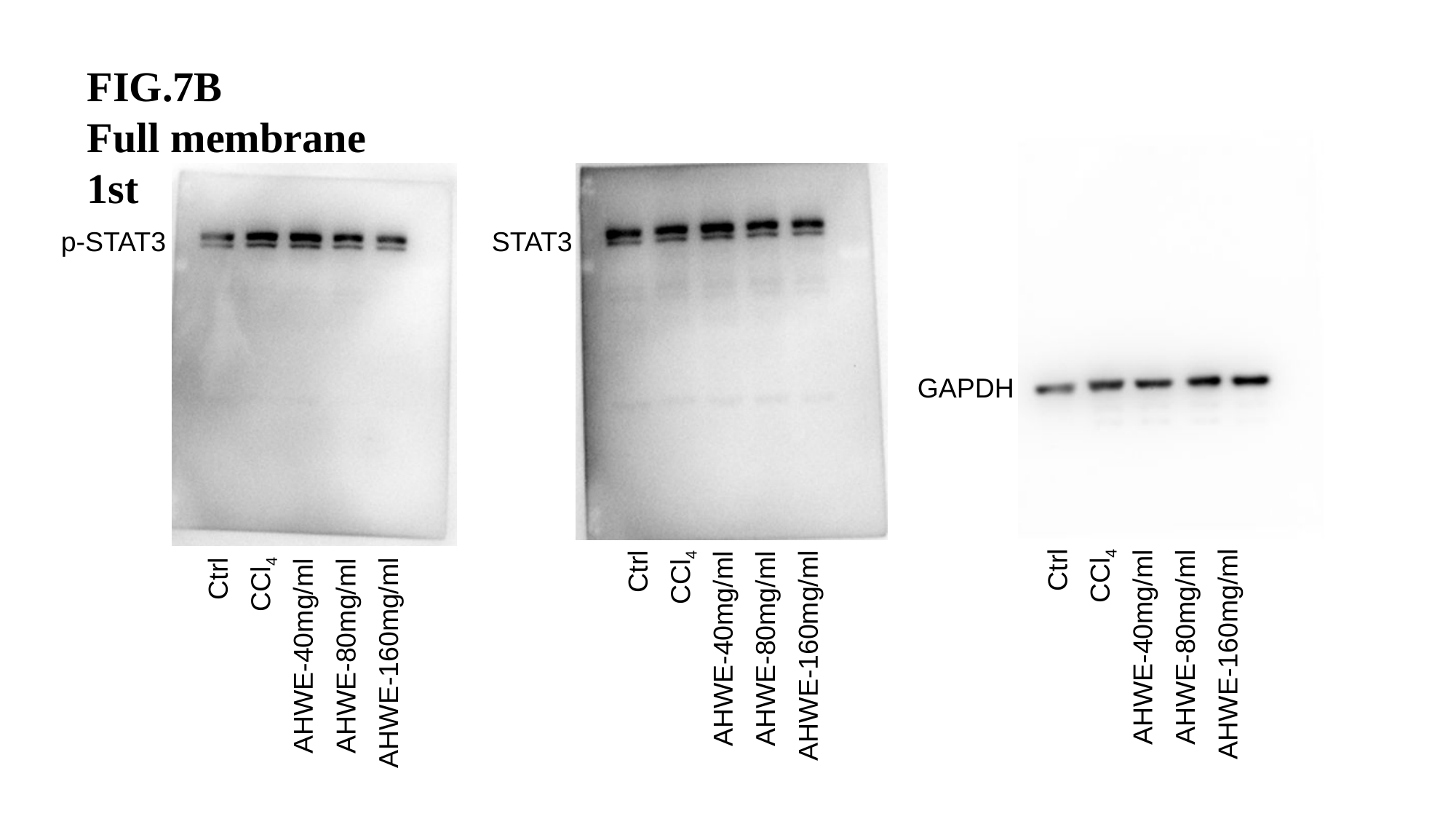

FIG.7B
Full membrane
1st
p-STAT3
STAT3
GAPDH
Ctrl
CCl4
AHWE-40mg/ml
AHWE-80mg/ml
AHWE-160mg/ml
Ctrl
CCl4
AHWE-40mg/ml
AHWE-80mg/ml
AHWE-160mg/ml
Ctrl
CCl4
AHWE-40mg/ml
AHWE-80mg/ml
AHWE-160mg/ml

## Slide 21
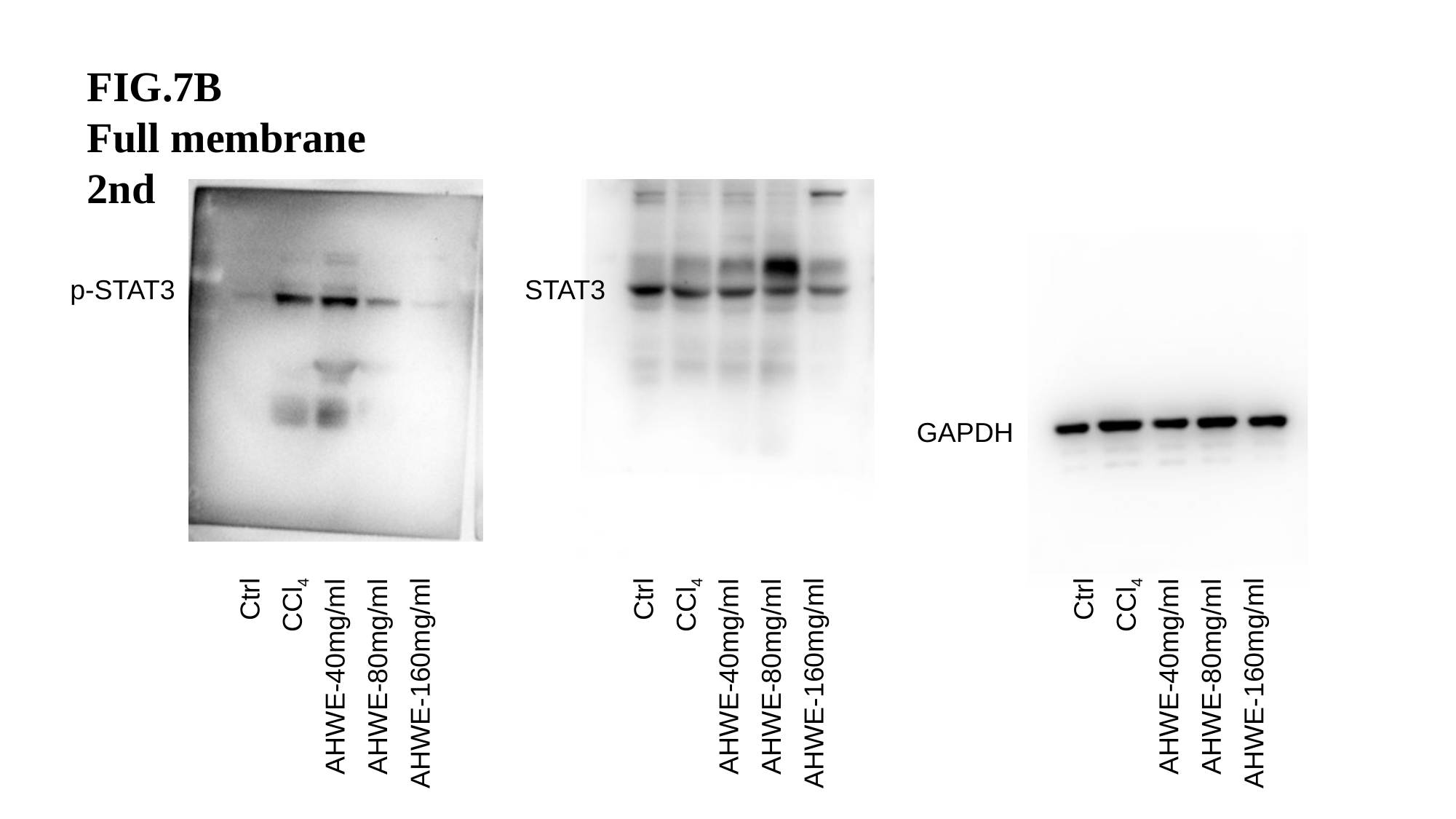

FIG.7B
Full membrane
2nd
p-STAT3
STAT3
GAPDH
Ctrl
CCl4
AHWE-40mg/ml
AHWE-80mg/ml
AHWE-160mg/ml
Ctrl
CCl4
AHWE-40mg/ml
AHWE-80mg/ml
AHWE-160mg/ml
Ctrl
CCl4
AHWE-40mg/ml
AHWE-80mg/ml
AHWE-160mg/ml

## Slide 22
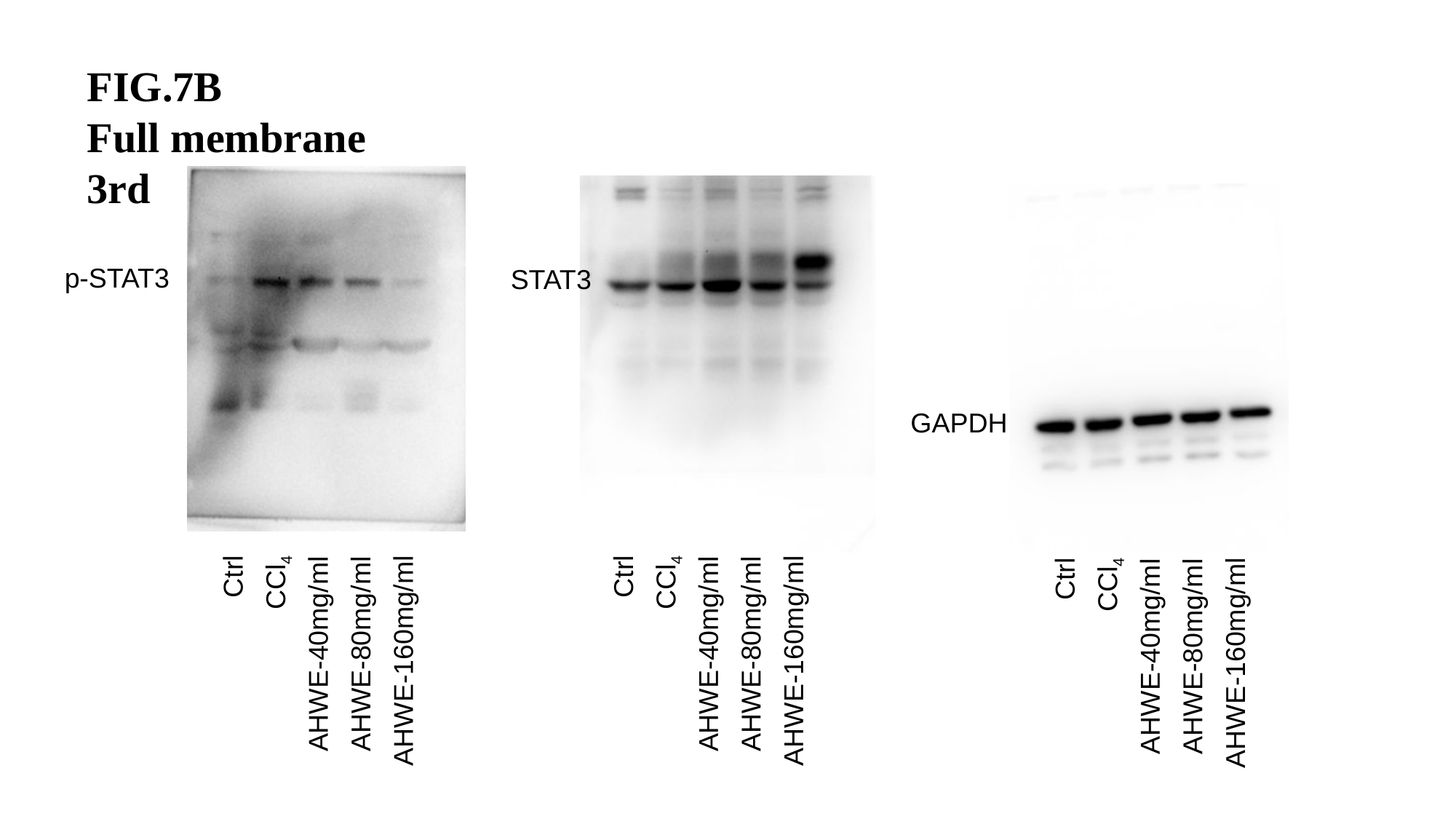

FIG.7B
Full membrane
3rd
p-STAT3
STAT3
GAPDH
Ctrl
CCl4
AHWE-40mg/ml
AHWE-80mg/ml
AHWE-160mg/ml
Ctrl
CCl4
AHWE-40mg/ml
AHWE-80mg/ml
AHWE-160mg/ml
Ctrl
CCl4
AHWE-40mg/ml
AHWE-80mg/ml
AHWE-160mg/ml
