# Supplementary material for: Yao medicine Amydrium hainanense suppresses hepatic fibrosis by repressing hepatic stellate cell activation via STAT3 signaling
Source: Front Pharmacol. 2022 Dec 14;13:1043022. doi: 10.3389/fphar.2022.1043022 (PMC9794994; doi:10.3389/fphar.2022.1043022)
Supplement: Supplementary file 3 [file Presentation2.PPTX]

## Slide 1
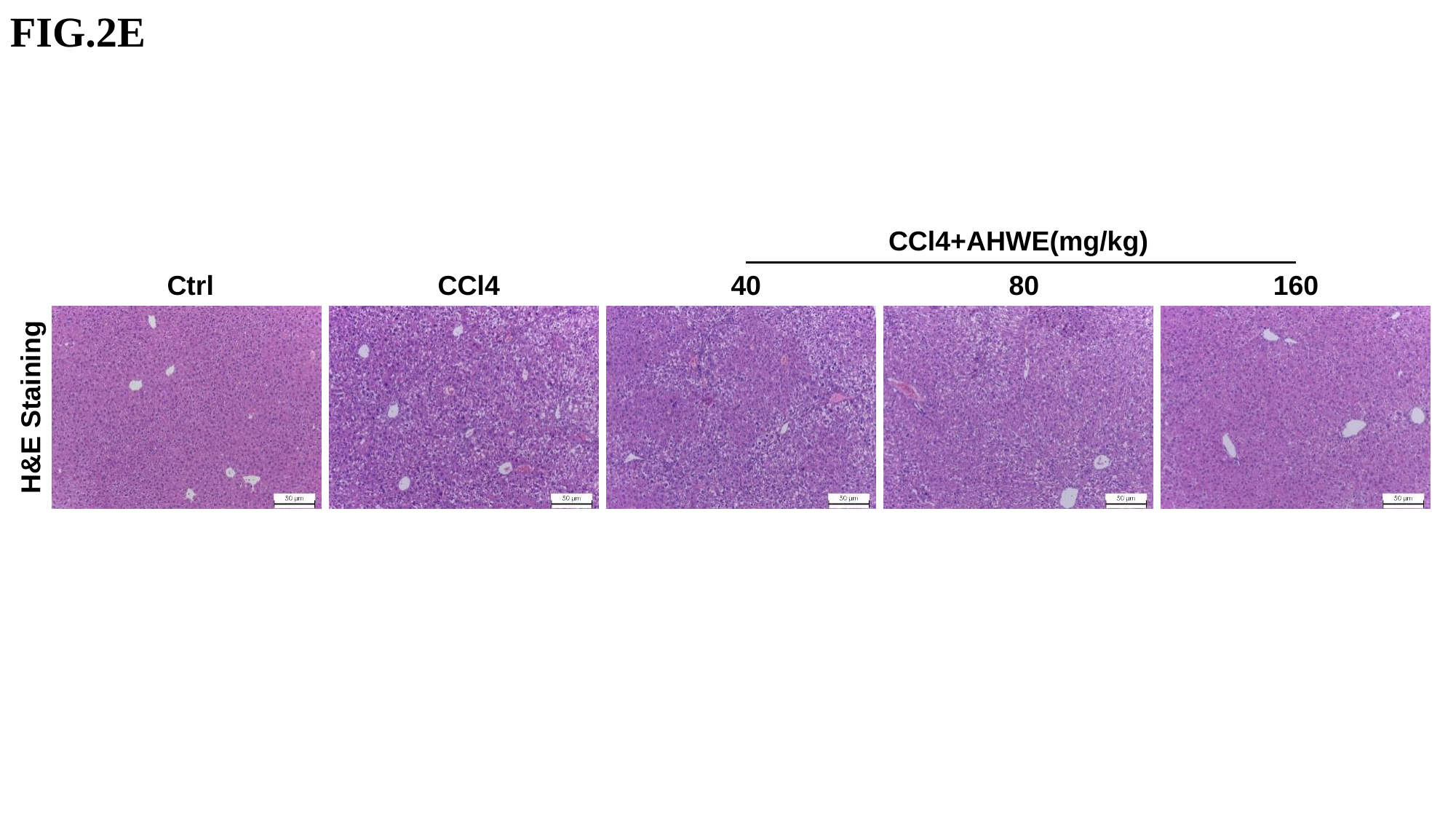

FIG.2E
CCl4+AHWE(mg/kg)
Ctrl
CCl4
40
80
160
H&E Staining

## Slide 2
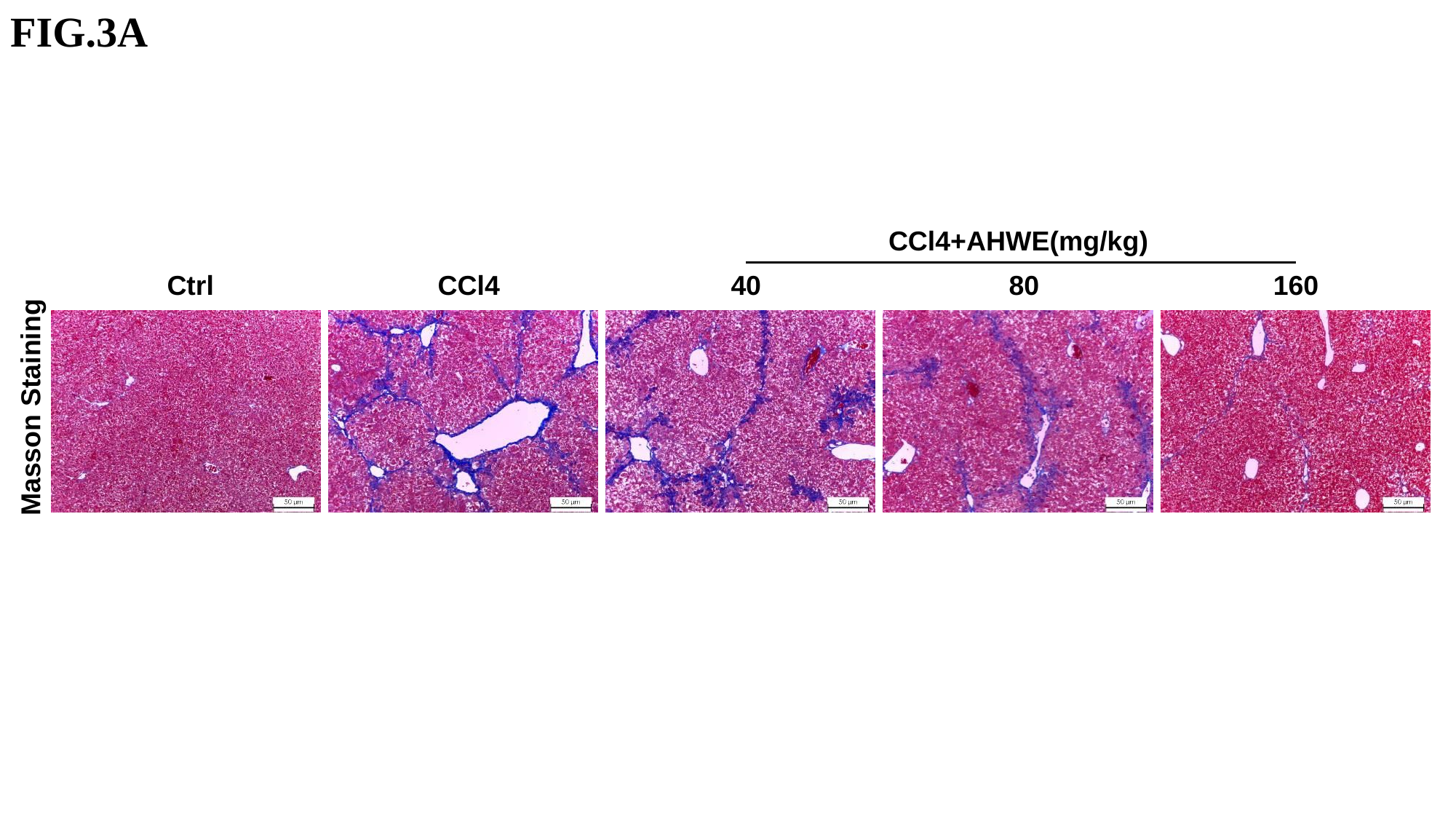

FIG.3A
CCl4+AHWE(mg/kg)
Ctrl
CCl4
40
80
160
Masson Staining

## Slide 3
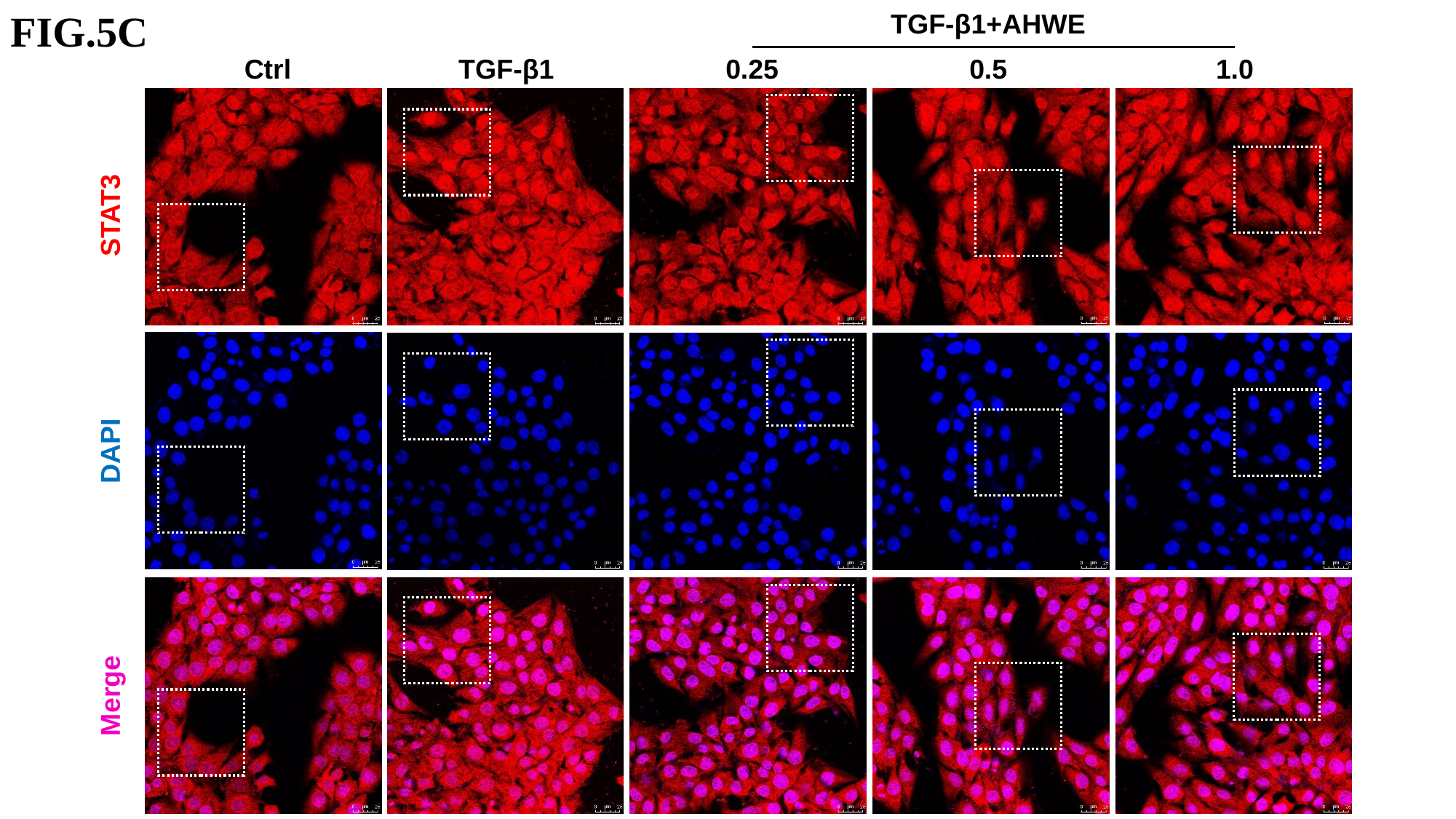

FIG.5C
TGF-β1+AHWE
Ctrl
TGF-β1
0.25
0.5
1.0
STAT3
DAPI
Merge

## Slide 4
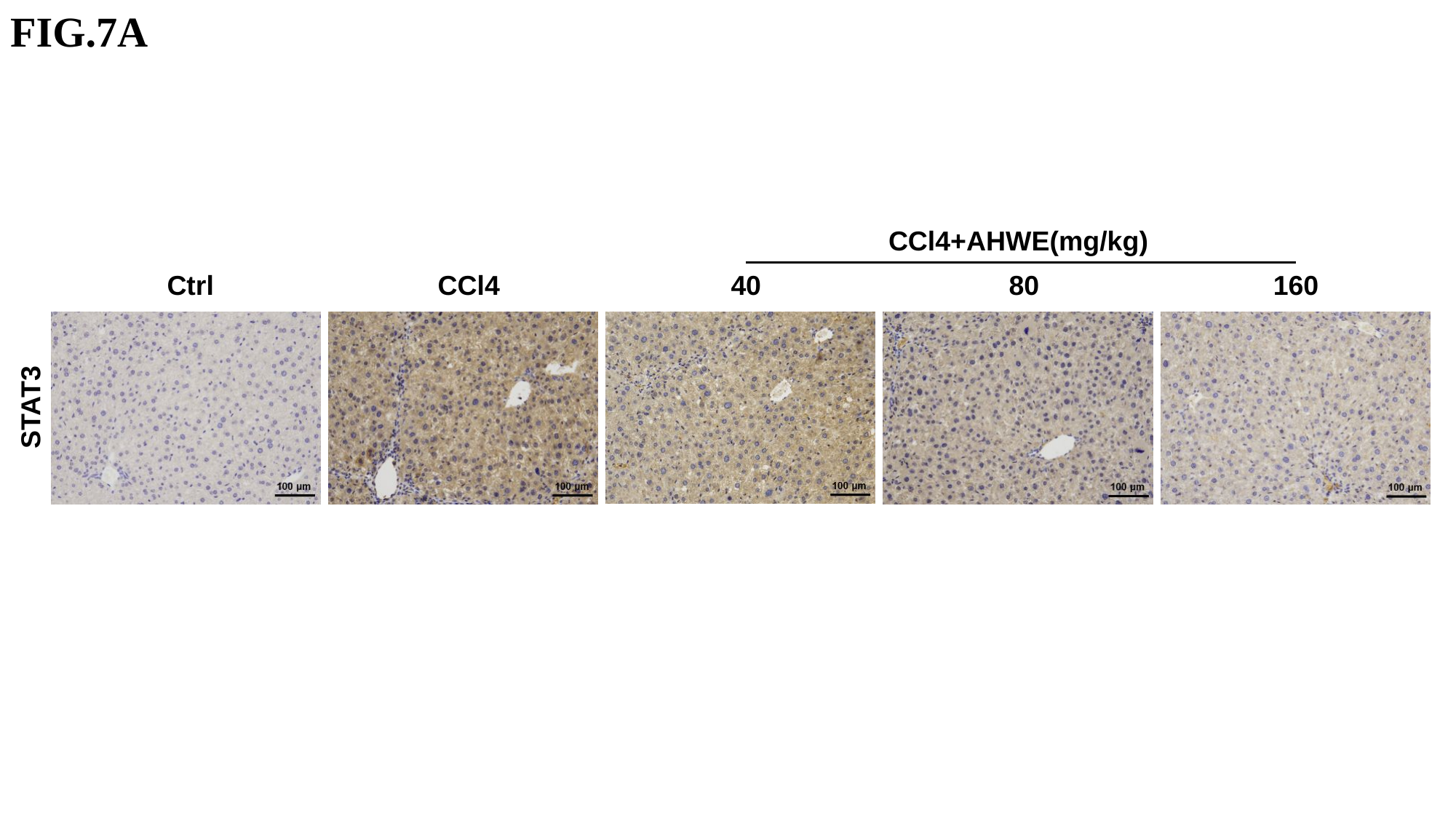

FIG.7A
CCl4+AHWE(mg/kg)
Ctrl
CCl4
40
80
160
STAT3
